# Engineering of oriented carbon nanotubes in composite materials

**DOI:** 10.3762/bjnano.9.41

**Published:** 2018-02-05

**Authors:** Razieh Beigmoradi, Abdolreza Samimi, Davod Mohebbi-Kalhori

**Affiliations:** 1 Department of Chemical Engineering, University of Sistan and Baluchestan, University Blvd., Zahedan 98167-45845, Iran,; 2 Innovation Center for Membrane Technology (ICMT), University of Sistan and Baluchestan, University Blvd., Zahedan 98167-45639, Iran

**Keywords:** arrangement and alignment, carbon nanotubes, composite materials, orientation

## Abstract

The orientation and arrangement engineering of carbon nanotubes (CNTs) in composite structures is considered a challenging issue. In this regard, two groups of in situ and ex situ techniques have been developed. In the first, the arrangement is achieved during CNT growth, while in the latter, the CNTs are initially grown in random orientation and the arrangement is then achieved during the device integration process. As the ex situ techniques are free from growth restrictions and more flexible in terms of controlling the alignment and sorting of the CNTs, they are considered by some as the preferred technique for engineering of oriented CNTs. This review focuses on recent progress in the improvement of the orientation and alignment of CNTs in composite materials. Moreover, the advantages and disadvantages of the processes are discussed as well as their future outlook.

## Review

### Introduction

**Figure 1:**
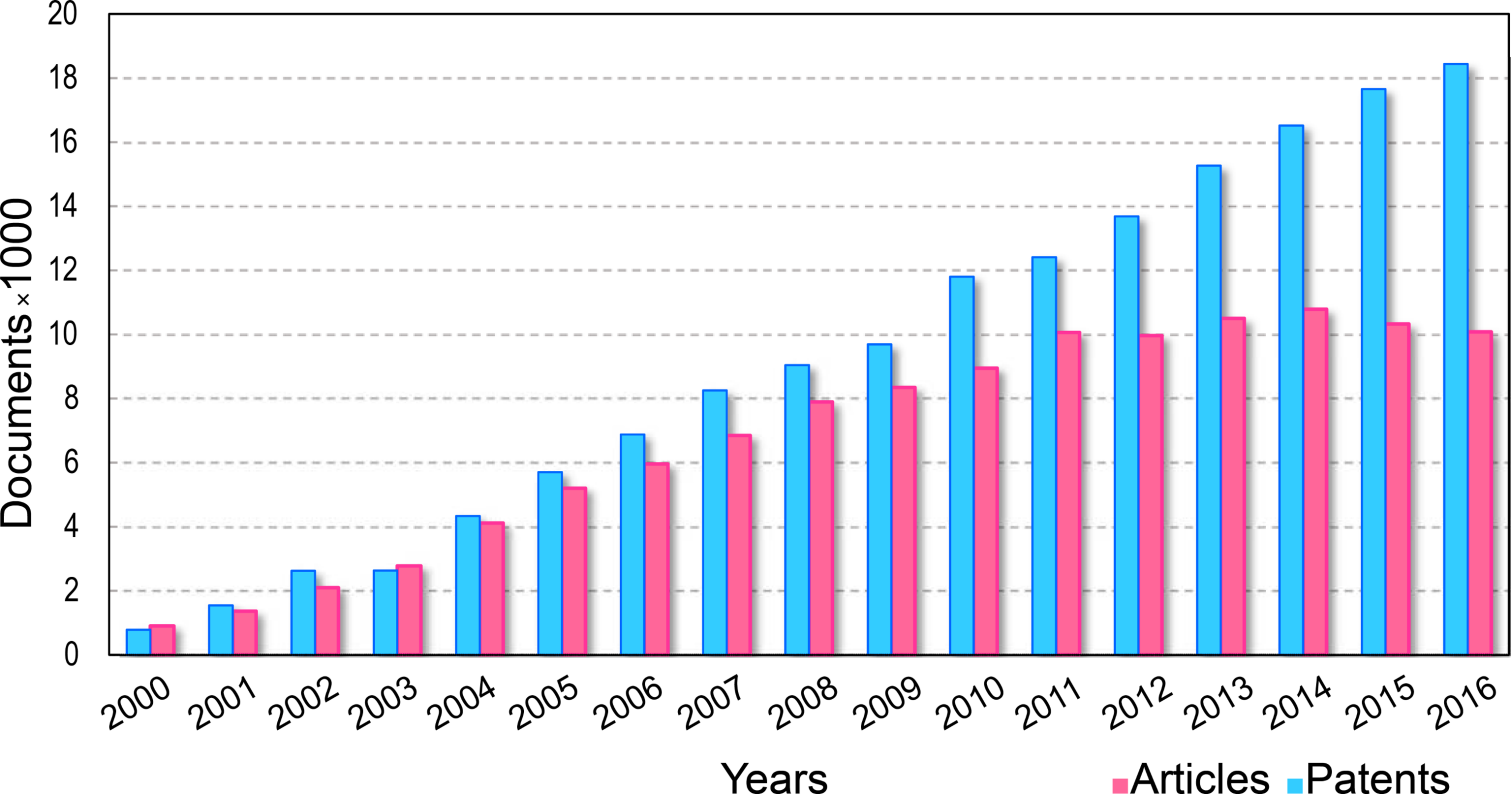
Number of papers and patents published in the past 16 years on the topic of CNTs. The numbers were determined based on a Scopus search spanning from 2000 to 2016 using the keywords: carbon nanotubes and CNT.

**Figure 2:**
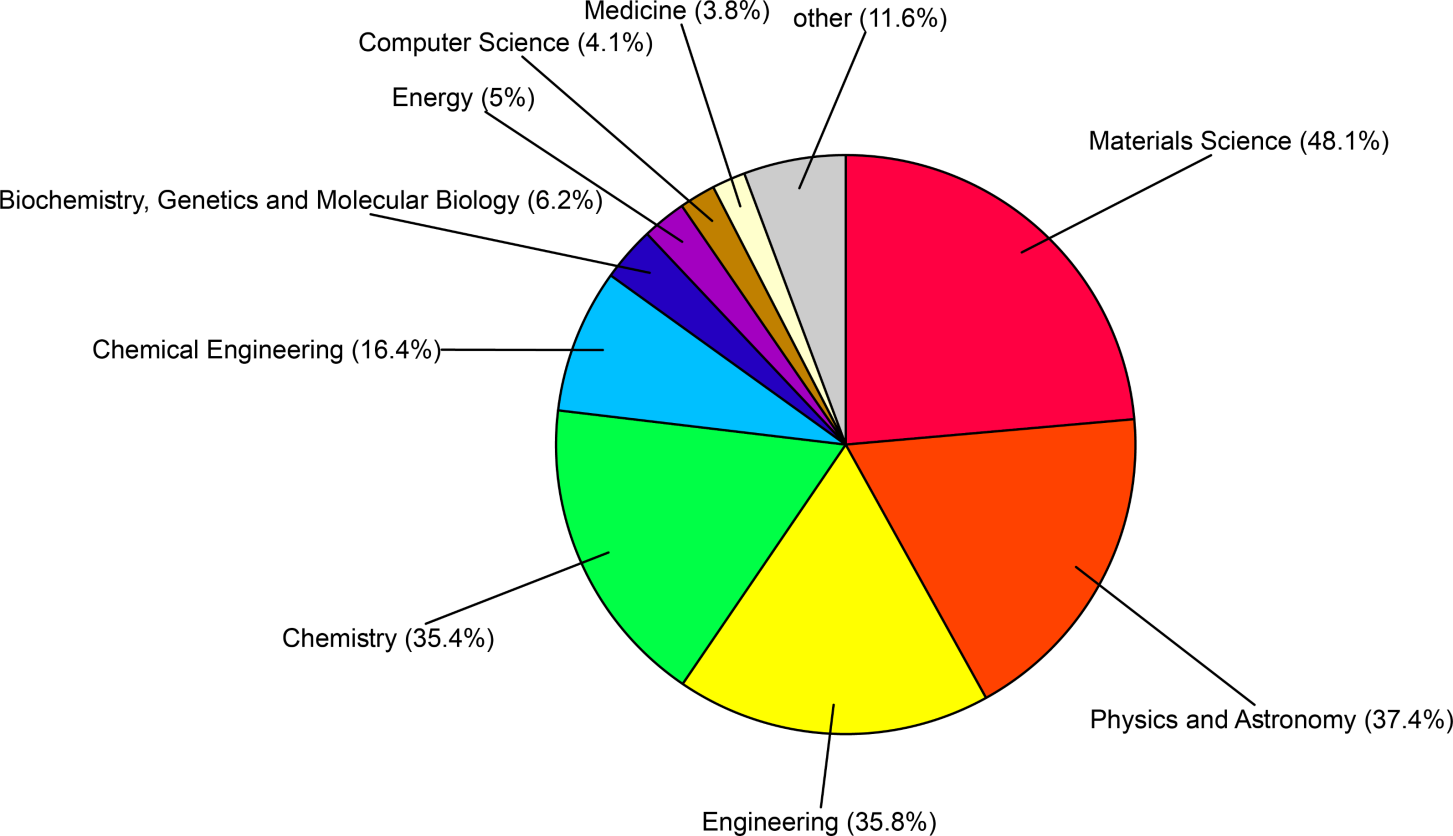
Subject areas of papers published in the past 16 years on the topic of CNTs. The numbers were determined based on a Scopus search spanning from 2000 to 2016 using the keyword carbon nanotubes.

Carbon is one of the most abundant elements comprising the world around us. Before 1985 graphite and diamond were the only known structural forms of carbon [1]. In 1991, Iijima discovered a new carbon structure which later became known as the carbon nanotube (CNT) [2]. Depending on the number of walls in the structure, CNTs are categorized as single-walled CNTs (SWCNTs), double-walled CNTs (DWCNTs) or multiwalled CNTs (MWCNTs). A SWCNT is the result of rolling a graphite sheet, and a MWCNT consists of many coaxial single-walled tubes nested inside each other. Considering the excellent mechanical and electrical characteristics of CNTs, interest in using them in industry is increasing every day. Various industrial applications of produced CNT composite materials, especially electronic, military, and new composites, has drawn the attention of researchers to this topic in recent decades [3–5]. Figure 1 and Figure 2 represent the growth rate of papers and patents and the fields of application of CNTs in the past 16 years, based on Scopus data, respectively.

As indicated, the areas of research regarding CNTs are vast and extend to several subcategories. In each subcategory issues such as structural uniformity, dispersion and stability, superficial defects, and intermolecular and composite interactions are very important [6–8].

Nowadays, the biggest challenge in using all the capabilities and exceptional properties of CNTs in the production of new materials is essentially the limitation of a lack of uniform structure which could be obtained by controlling their sorting and orientation. Because the properties of CNTs vary according to size and shape (diameter, length and whether they are open- or close-ended), their arrays and arrangement in composite materials may lead to different properties.

For example, in their recent research, Nam et al. have shown the effect of diameter in a composite material containing aligned CNTs [9]. This study showed that composites fabricated with a smaller average diameter have significantly better mechanical properties. Gulotty et al. have reported that CNTs with a longer and larger diameter more efficiently improve the thermal conductivity of polymer composites [10].

Furthermore, it has been shown that for biological applications the CNT diameter and length are critical parameters in protein corona formation and biocompatibility [11–12].

In another research investigating the effect of aligning CNTs in composite material structures, a remarkable improvement in the electrical properties of CNT composites was observed, as compared to their random placements [13]. In recent research reported by Lee et al., two factors, the CNT length and their arrangement, were introduced as important characteristics affecting electron transport properties in the matrix of composite materials [14].

CNTs are excellent alternatives to metal oxides for metal-free catalysis [15] or in synergy with metal oxides [16–17], especially for sustainable energy applications [18–19]. Because of their electronic properties, CNT composites offer unmatched opportunities for conductive tissue regeneration [20], particularly if alignment, and thus 3D anisotropy, is achieved for the engineering of cardiac [21], muscle [22] and nerve tissues [23]. Finally, CNT properties can be fine-tuned upon chemical functionalization. However, the latter characteristic needs to be well-balanced in order to prevent degradation of the CNT properties [24–26].

The results of these studies and similar reports indicate that methods by which the arrangement of CNTs can be controlled and adjusted are of great importance. In fact, if nanocomposites with an adjusted arrangement and desired distribution of CNTs could be produced, then our expectations about the properties of CNT nanocomposites could be fulfilled [14,27–30].

In terms of processing techniques, the arrangement and alignment of CNTs in a matrix of composite materials are divided in two categories. In the first category alignment is arranged during growth, where the regular placement of catalyst nanoparticles on the substrate leads to growth of CNT masses in regular rows. In the second category, the arrangement occurs after growth. There have been several review articles written about the alignment methods of CNTs [6,31–35]. However, most of these focus on the synthesis of aligned CNTs, and the number of methods reporting the arrangement of CNTs after their production is limited.

In this paper, arrangement techniques utilized after the growth of CNTs, regardless of the orientation of the CNTs, are specifically reviewed. In these techniques, CNTs are added using various methods as an additive to the structure of the desired material to attempt to align the CNTs in the network of composite material. The uniform alignment is obtained to achieve the expected desirable and unique properties for CNTs. Furthermore, methods for characterization and evaluation of CNTs (regarding their arrangement) are presented and discussed.

### Post-growth sorting of CNTs in a composite material structure

As mentioned above, the methods for arranging CNTs are divided into two main categories: orientation during growth (in situ) and post-growth (ex situ). In post-growth orientation processes, the CNTs are initially produced using conventional methods. Then, by applying a distinct process, they are arranged for a specific purpose. Compared with in situ methods, ex situ methods do not have limitations in the production of CNTs, such as restriction on the substrate type and processing temperature, and also the variety of their final product is much greater. In the following, the post-growth sorting methods are discussed in more detail.

#### Carbon nanotube/polymer film stretching

**Figure 3:**
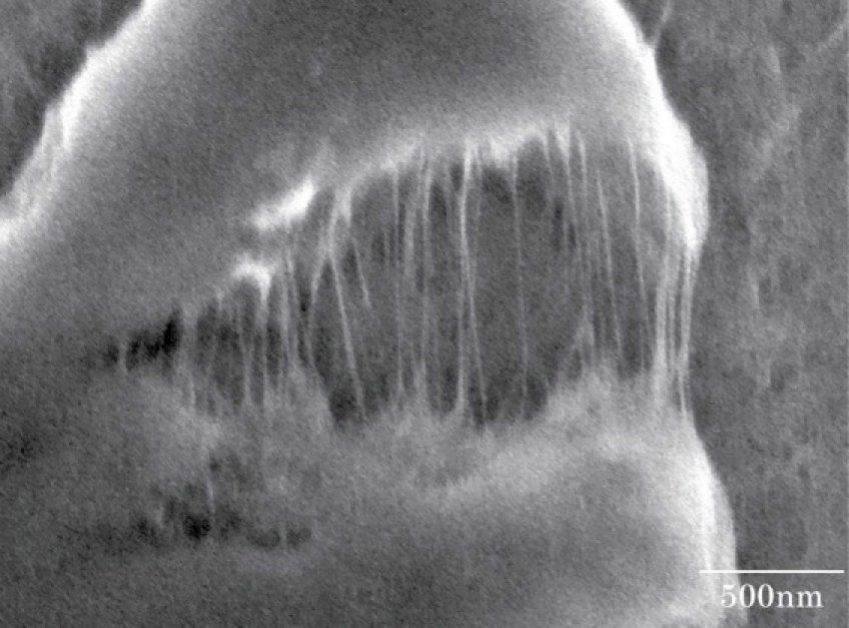
SEM image of a CNT/polymer film. The oriented CNTs are indicated in the fractured part of the composite. Reproduced with permission from [33], copyright 2009 Versita Warsaw.

By dipping a polymer substrate into a well-dispersed CNT solution a thin layer of CNTs is set on the polymer, and then the clamped opposite edges of the substrate are uniaxially stretched. A relatively warm air flow (approximately 60–100 °C) is used during the stretching process to keep the polymer soft. Then, a very small but steady twisting action is performed by hand. During the stretching process, the CNT direction is changed under the elastic field from the polymer matrix [33] (Figure 3). The stretching ratio depends on the length ratio of the thin layer before/after stretching. Yao et al. prepared a MWCNT/polyvinylidene fluoride (PVDF) nanocomposite at a relatively high concentration of CNTs and aligned them inside the polymer matrix by stretching. They reported that a high degree of alignment was achieved and the composite dielectric properties changed upon CNT alignment [36].

Although this method is relatively simple, and some polymers such as polyethylene can be stretched more than 30 times without failure, its application is limited to the laboratory because of the inhomogeneous stretching ratio at the edge of films need manual operation and a skillful operator [17].

#### Fracture stretching

**Figure 4:**
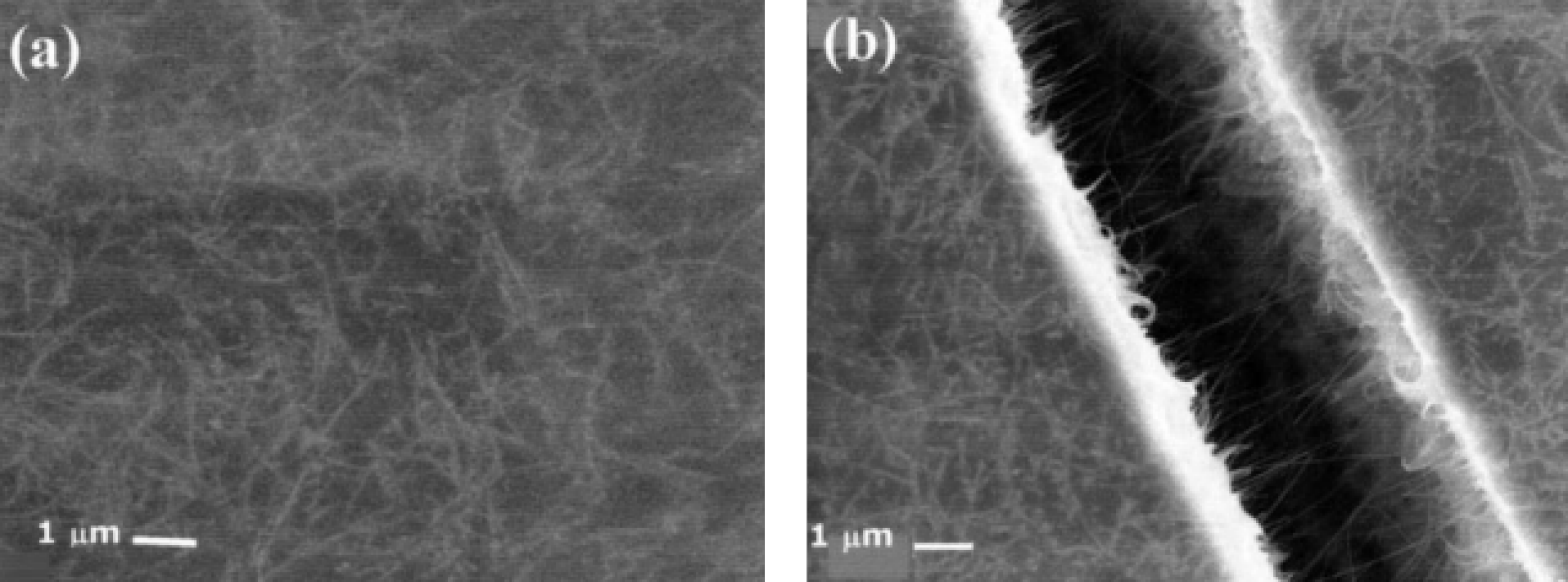
SEM images of a polystyrene (PS) composite film containing 25 wt % CNTs. (a) Random networked CNTs on the film surface and (b) oriented CNTs by fracturing, reproduced with permission from [37], copyright 2002 AIP publishing LLC.

An ultimate case of stretching is known as fracture and occurs when a hard polymer composite comprising CNTs, such as polyurethane (PU) or polystyrene (PS), is stretched too far. Fracture occurs and the oriented CNTs are formed in the fracture gap as shown in Figure 4. Since CNTs have the desired electronic properties and the method is easy to perform, this technique might be suitable for making CNT electronic devices such as field emitters [6,27–28,31,33,37–39].

**Figure 5:**
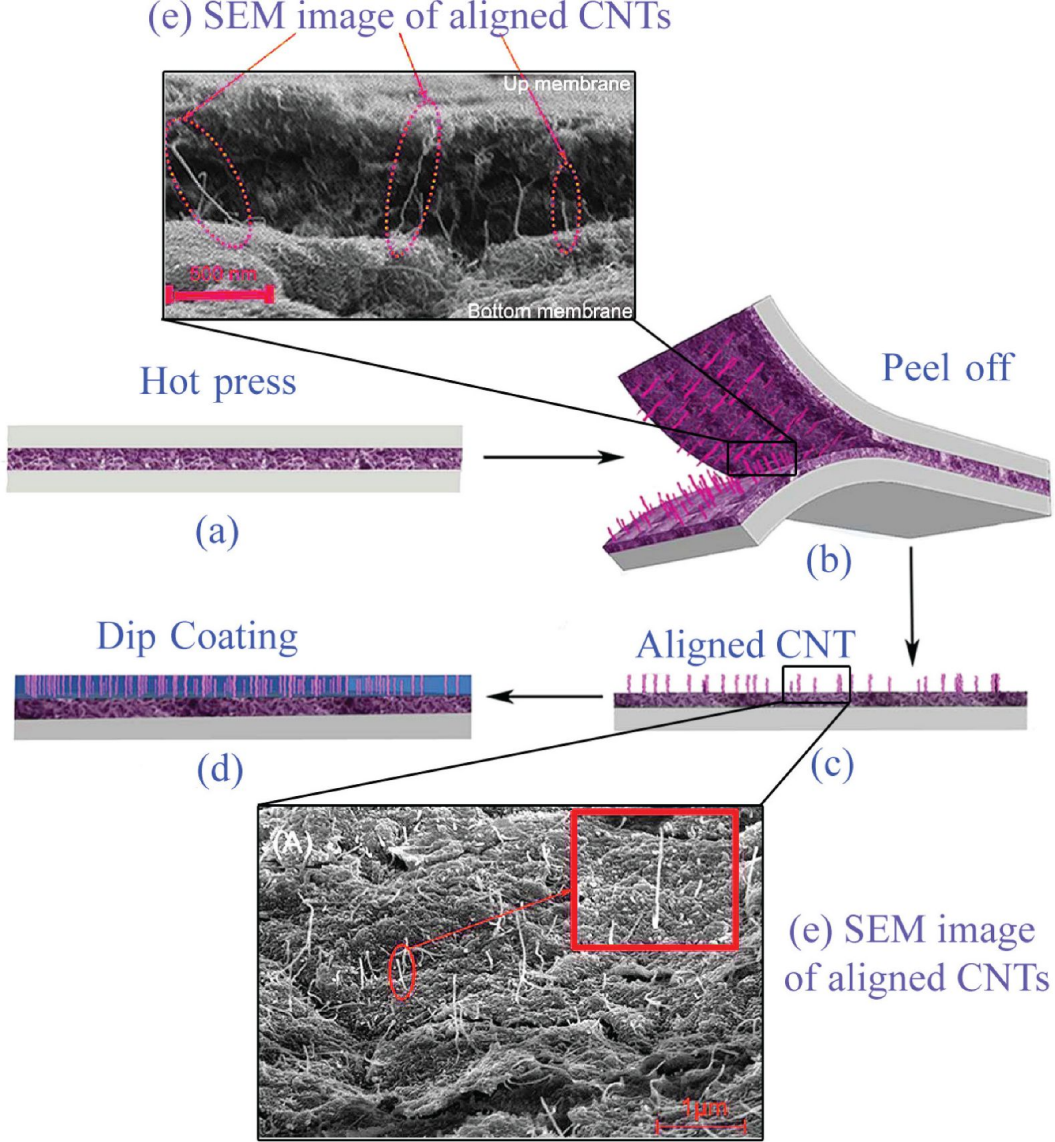
Fracture stretching method containing two steps, hot press and peel off, to align CNTs. (a) The hot press of the polysulfone (PSF) layer on the composite film of embedded CNTs on a nylon polymer membrane by spraying suspension until dry, (b,c) peel off the PSF layers from the nylon layer leading to the aligned CNTs, (d) mask CNT composite film with cellulose acetate (CA) casting solution, and (e) SEM images of aligned CNTs. Adapted with permission from [40], copyright 2013 Nature Publishing Group.

In recent research, fracture and stretching processes have jointly been used to align CNTs in a controlled manner [40]. In this process, functionalized CNTs were initially well-dispersed in water using ultrasound and then were sprayed on a nylon membrane or the polymeric membrane was directly dipped in a suspended mixture of CNTs. By using vacuum evaporation, the water content of the composite was ultimately evaporated. The latter CNT–polymer composite was then covered with a polysulfone (PSF) layer. The resulting composite was then pressed for 10–20 seconds at a temperature of 100–120 °C and pressure of 106 Pa. Lastly, the composite sheet was carefully peeled off to form two uniform layers of PSF/nylon/CNTs (Figure 5). In fact, compression under heat treatment leads to a strong interaction between the membrane surface and CNTs. Because of the angle of the gap between the two layers and the simultaneous effects of the shear force and mechanical tensile stretch, a slight drag force pulls the CNT in the vertical direction. However, the obtained free CNTs are certainly not vertical relative to the surface of layers. Although the small length of the tubes limits the angle of the gap, it also leads to a very small angle deviation in the direction of the shear force. In principle the tubes were not constrained to be quite vertically oriented, but a microscopic view showed satisfactory alignment. To keep the CNTs aligned, the top layer of the CNT/PSF membrane could be coated by cellulose acetate (CA) solution.

#### Frictional orientation

The frictional orientation method is known as the doctor blade (DB) technique or tape casting. This technique is widely used to produce a variety of thin layers and coat substrates with wet films. A blade or a spiral film applicator is generally used to rub the layers. When a polymer layer including CNTs is rubbed in a certain direction while being softened by a heating process, elastic forces tend to orient and align the CNTs. Although this method can be performed automatically, due to damages that may be exerted on the polymer layer, manual alignment is preferred [41].

**Figure 6:**
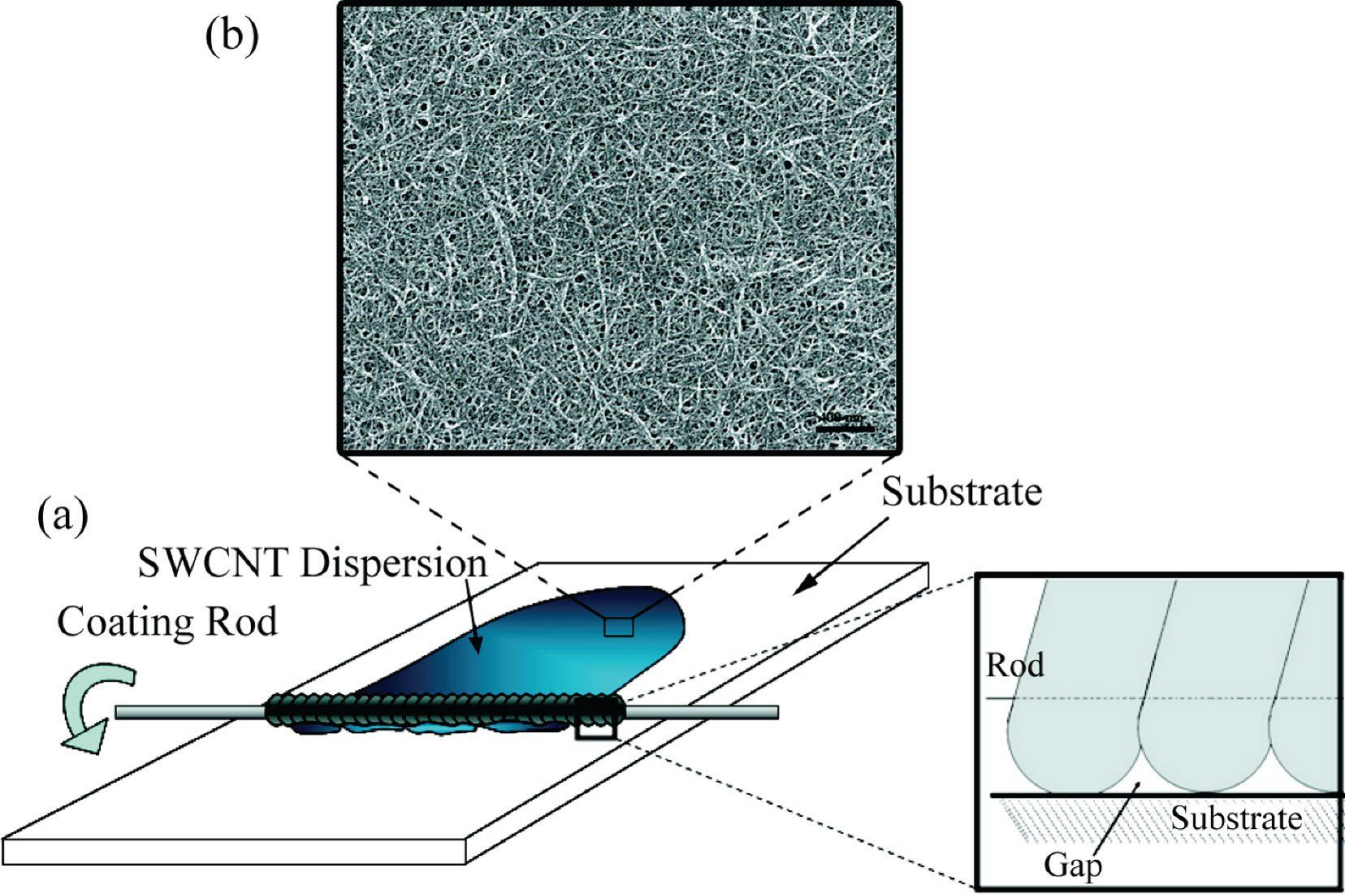
Schematic setup of a spiral film applicator (a) and SEM image of arranged CNTs (b). Adapted with permission from [42], copyright 2009 American Chemical Society.

Based on the fundamental principles of this approach, another method for making thin films of CNTs has been developed using the same tools. Fluid containing CNTs is spread on a solid substrate using a spiral film applicator. The surface tension and viscosity of the fluid are two important factors affecting the final result of deposition on the substrate. In Figure 6a, a schematic of a designed tool for this purpose is shown [42].

#### Carbon nanotube/fibrous composites

Over the last century, fibrous structure nanocomposites have been extensively considered due to their desirable physical properties [43]. In this regard, CNT/fibers have obtained a special spotlight because of their possibility to be arranged in a controlled way, the dependency of bulk electrical and mechanical properties of composites on the characteristics of the CNT, and the ensemble and production ability of the fibers in semi-industrial quantities [44]. The two methods commonly used to make CNT/nanofiber are described below.

**Figure 7:**
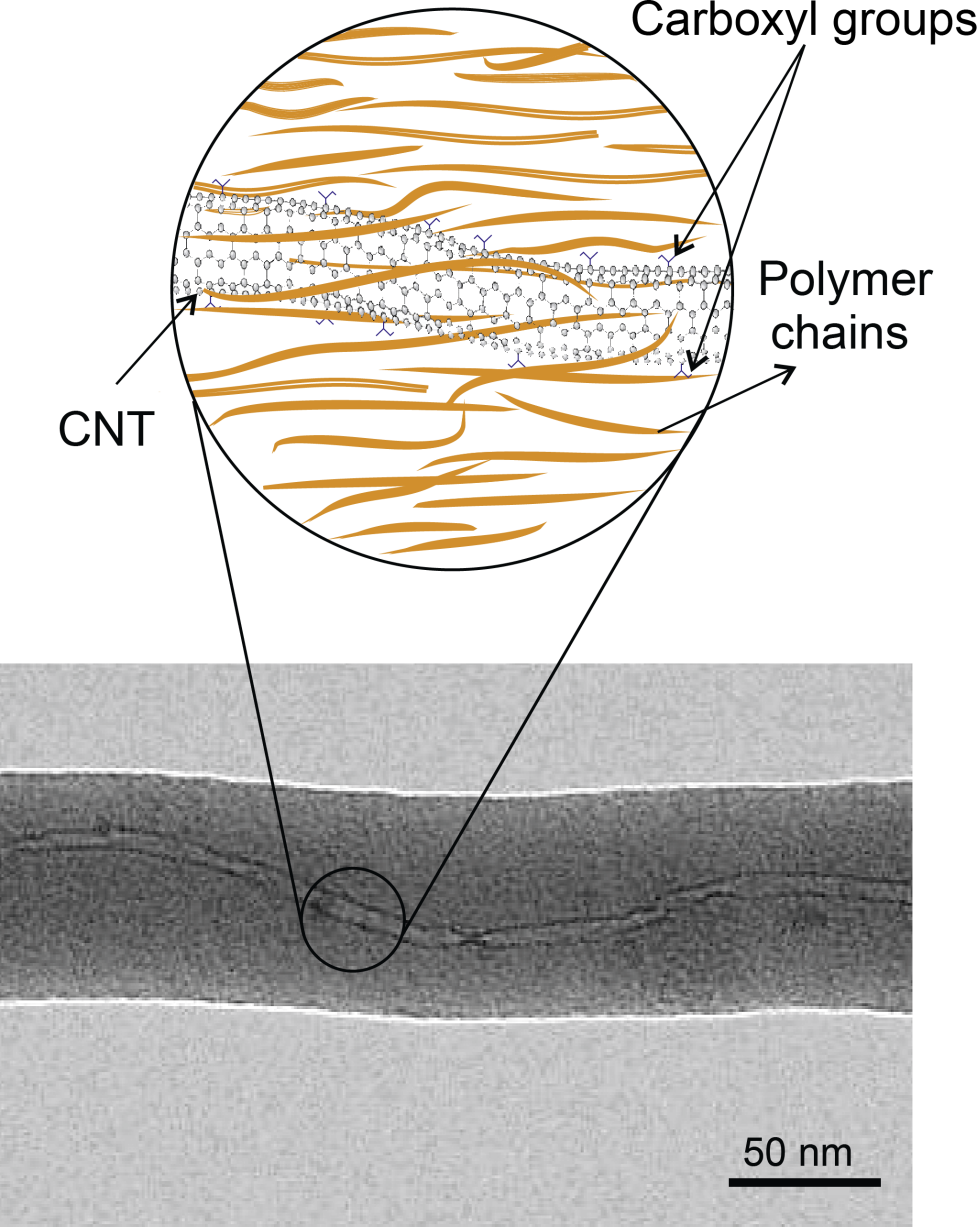
Schematic structure of an aligned CNT in a polymer nanofiber. TEM micrograph, adapted with permission from [48], copyright 2003 American Chemical Society.

**Electrospinning:** Electrospinning (ES) can be used to produce fibers from a viscous solution of polymer/CNTs, it is also employed for aligning CNTs in the fibers. In this method, a high voltage DC current (about 25 kV) is used between a charged polymer and a metallic collector to produce continuous filaments. Experiments revealed that the functionalized CNTs are aligned in the direction of the axis of the nanofiber polymers [45–47]. Figure 7 shows a TEM image of an orientated CNT embedded in a polymeric fiber.

As the orientation of the fibers also orients the CNTs in the same direction, in ES, the design of the collector plays the key role in the regular arrangement of the fibers and in enhancing the desired composite properties [49–52]. For example, Park et al. described how better alignment and mechanical and actuating performance of CNT/PVDF ES nanofibers was achieved by changing the drum collector parameters [51]. Their results indicated that the mechanical properties were improved up to 300% in the arranged direction.

**Figure 8:**
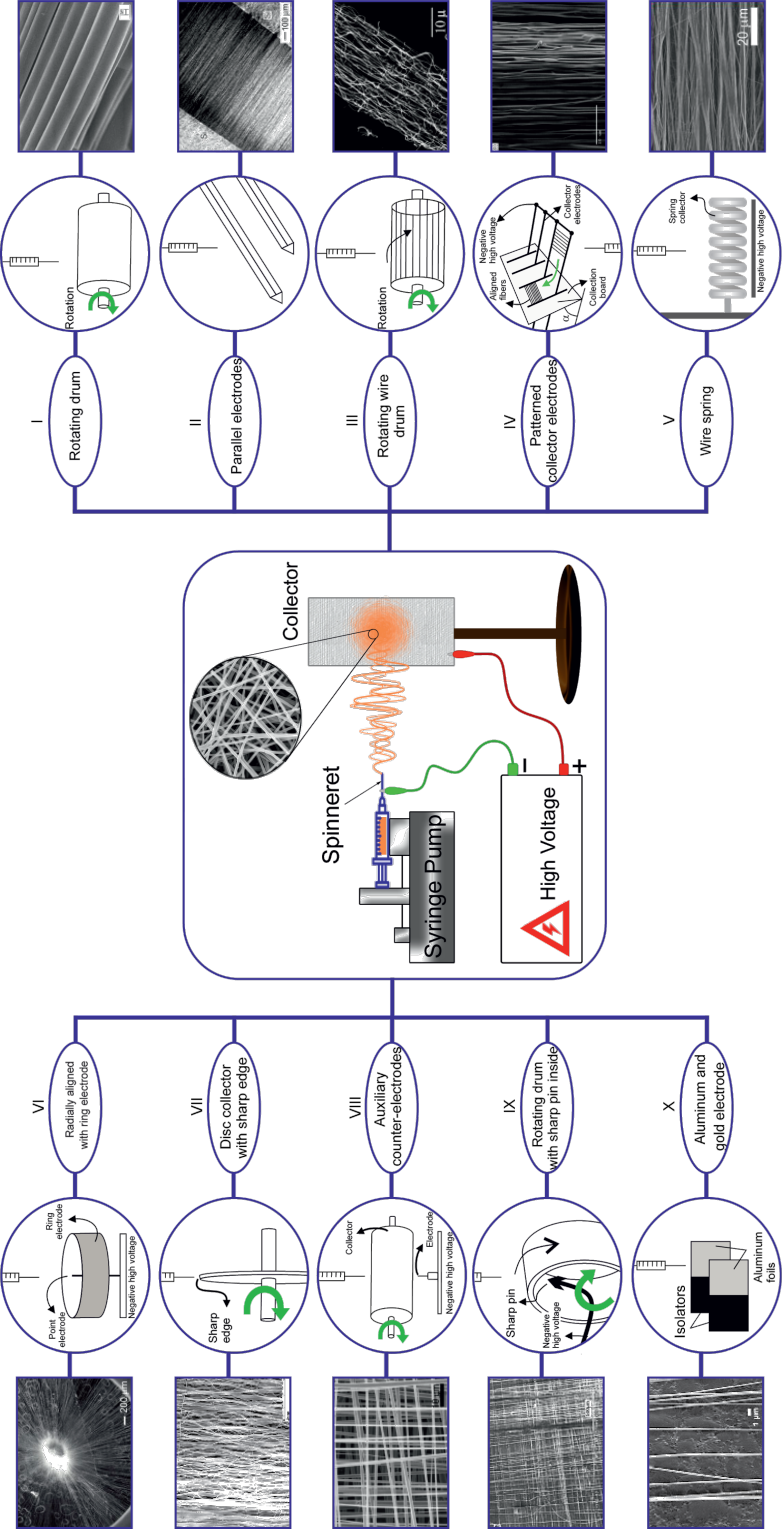
An electrospinning workflow illustrates a standard electrospinning setup, including power supply, syringe pump and collector plate and other collectors developed to collect oriented fibers. (I) [50,53–55], (II) [56], (III) [57], (IV) [58], (V) [59], (VI) [60], (VII) [61–62], (VIII) [63], (IX) [64], (X) [65]. SEM images: (I) Reproduced with permission from [55], copyright 2005 American Chemical Society, (II) Reproduced with permission from [56], copyright 2003 American Chemical Society, (III) Reproduced with permission from [57], copyright 2004 American Chemical Society, (IV) Reproduced with permission from [58], copyright 2008 AIP publishing LLC, (V) Adapted with permission from [59], copyright 2015 American Chemical Society, (VI) Adapted with permission from [60], copyright 2010 American Chemical Society, (VII) Reproduced with permission from [62], copyright 2009 American Chemical Society, (VIII) Reproduced with permission from [63], copyright 2008 American Chemical Society, (IX) Reproduced with permission from [64], copyright 2004 AIP publishing LLC, (X) Reproduced with permission from [65], copyright 2006 AIP publishing LLC. It should be noted that the electrospinning method is also appropriate for commercial applications due to its high throughput [66–69].

A standard setup of an ES device, including power supply, syringe pump, and collector plate, is indicated in the central part of Figure 8. The upper and lower sections of Figure 8 show other types of collectors developed to collect the oriented fibers.

**Figure 9:**
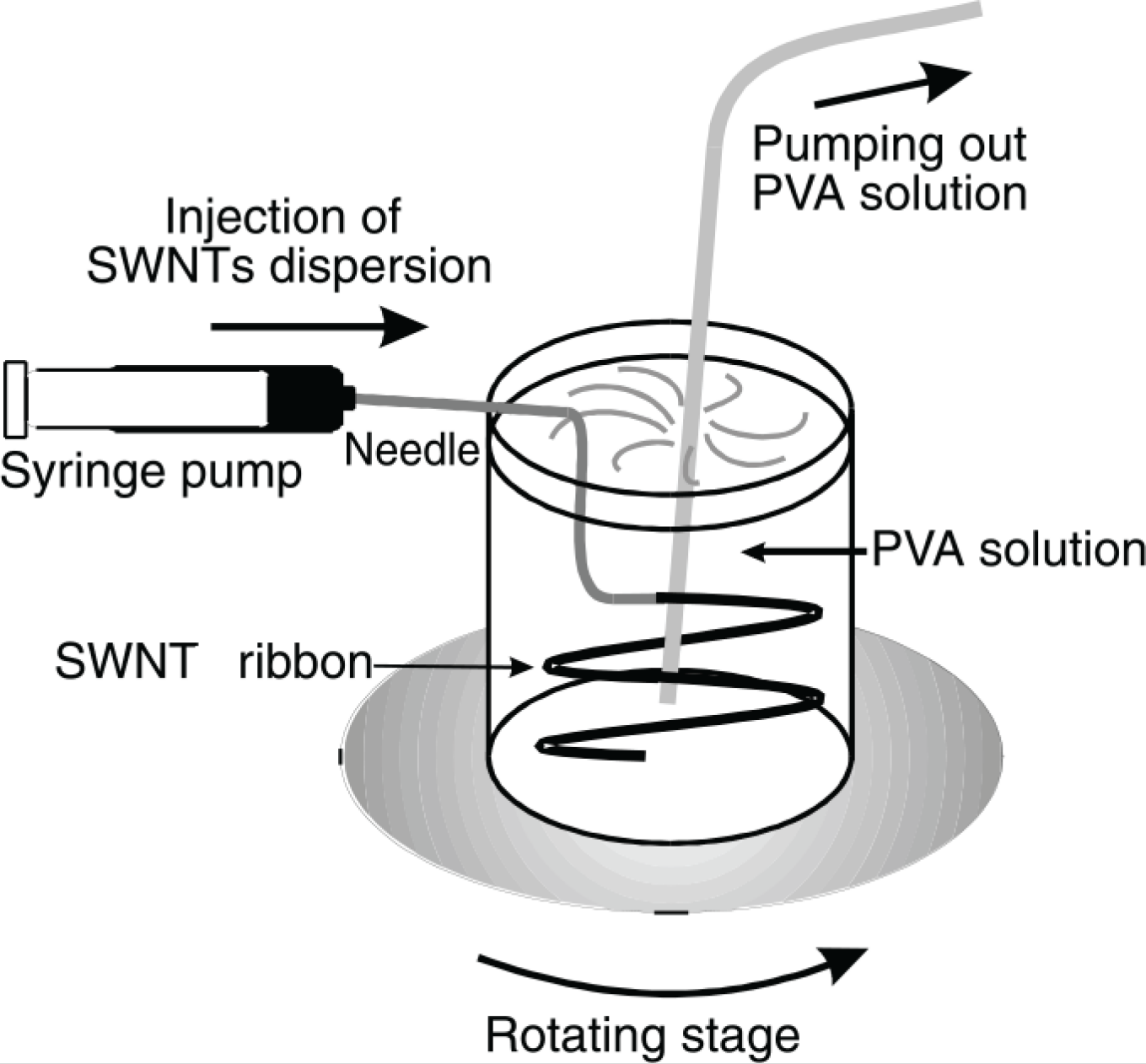
Schematic of the experimental setup for the wet spinning technique, reproduced with permission from [70], copyright 2000 The American Association for the Advancement of Science.

**Fiber Drawing:** Coagulation spinning is another method recommended for producing concentrated and aligned composite nanomaterials. In this method SWCNTs are initially dispersed in an aqueous solution of poly(vinyl alcohol) (PVA) and are stabilized using surfactants. By applying the wet spinning method, a web of nanocomposite material fibers is produced where the arrangement of CNTs and sorting of nanofibers are done at the same time, as shown in Figure 9 [70].

Recently, direct spinning to a vertical chemical vapor deposition (CVD) synthesis zone has also been studied and is under development to produce CNT fibers and ribbons [44,71]. In a vertical CVD reactor, the CNT fibers are fabricated by drawing an aerogel of CNTs from the gas phase during growth [44]. The drawing orients the CNT bundles and can be combined with other techniques to fabricate diverse composites. The experimental works indicate the ability of the method to produce continuous CNT fibers with a high degree of orientation on a large scale. However, catalytic impurities are still the biggest challenge of this method [72].

#### Spray winding and layer-by-layer deposition

Two of the latest controlled methods to produce composite polymer nanomaterial/CNTs are spray winding [73–76] and layer-by-layer deposition (LBL) [77–83].

Spraying [84–86] and electrospraying [87–92] are efficient methods to create a homogeneous layer of polymer liquid on the winding mandrel. Because of the simplicity and adjustability of the process and potential for use of a wide range of materials, spraying can be combined with other methods to fabricate composite materials.

**Figure 10:**
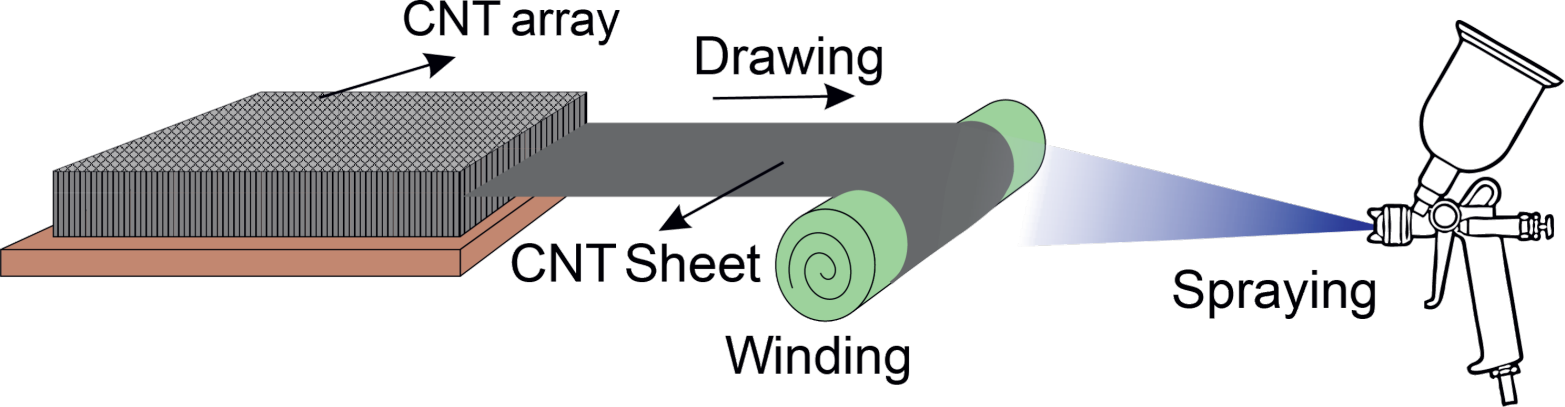
Spray winding apparatus. Step 1. Stretching the CNT array by passing along the stationary rod. Step 2. Winding onto a rotating mandrel. Step 3. Fixing with a matrix spray.

In this method, a sheet of CNTs is produced by chemical vapor deposition (CVD) on a SiO_2_/Si substrate that is coated with a very thin layer of iron as a catalyst. The CNT rows have been grown perpendicular to the substrate so that they form a completely continuous sheet, resulting in a very elastic structure [93]. Then this elastic sheet is placed on a mandrel and small droplets of solution are deposited by spraying a dilute solution of a polymer on the CNT sheet during controlled rotation of the mandrel. As a result, a layer of polymer/CNT composite material is formed as shown in Figure 10. Finally, this composite layer is compressed between two hot plates to remove air bubbles and enhance the bond between the polymer and carbon nanotube. This method is highly regarded because of the simplicity of the manufacturing process, industrial scalability and controllability of CNTs [94]. Although the alignment of CNTs occurs during their growth, in the spray winding method, the main orientation of the CNTs in the matrix of the composite material is carried out in a separate mechanical method. That is why this method is classified as an after growth orientation method rather than during growth alignment.

**Figure 11:**
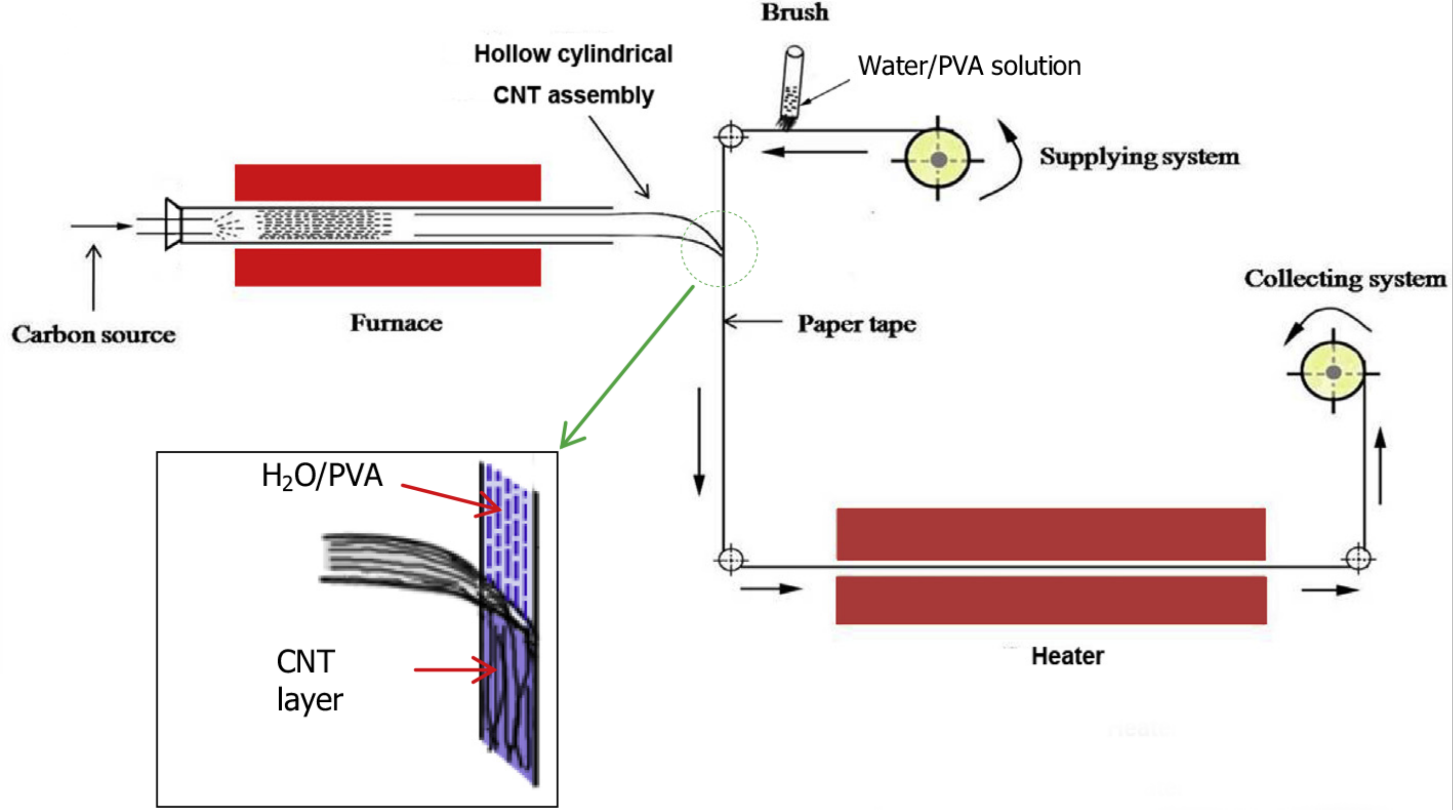
Schematic of the layer-by-layer deposition process. Step 1. Wetting a paper tape with water/poly(vinyl alcohol) (PVA). Step 2. Introducing the assembled CNT layer in the reactor. Step 3. Winding up after drying. Reproduced with permission from [78], copyright 2015 Elsevier.

In the layer-by-layer deposition method, the substrate is alternately and repeatedly dipped into an aqueous solution of functionalized targeted material, and in accordance with the functionalized material, controlled layers are deposited on the substrate. This method can be used to produce very thin and coherent layers, under mild conditions, in a wide range of composite materials (Figure 11).

**Figure 12:**
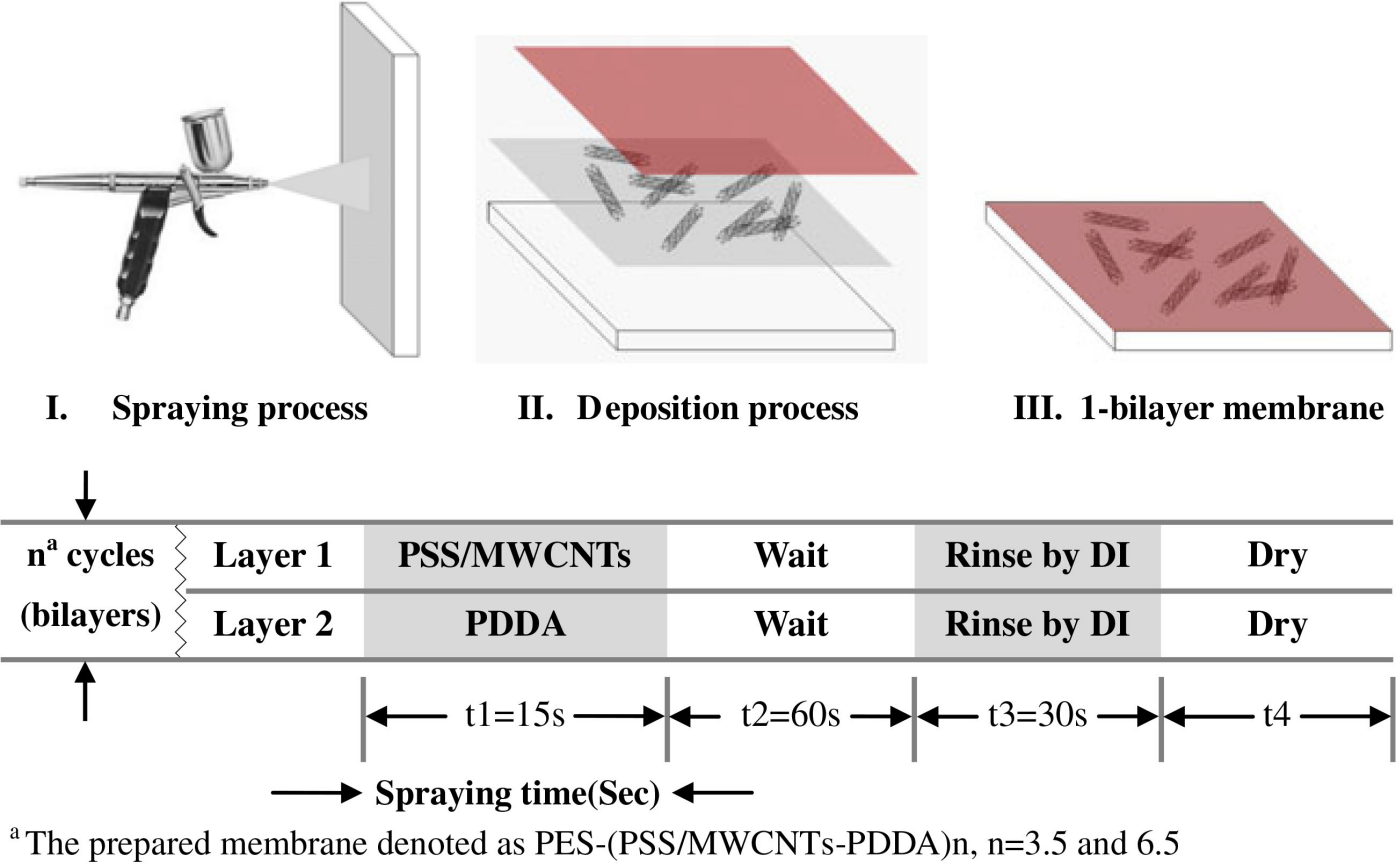
Layer-by-layer technique to fabricate an ultrathin CNT composite membrane. Reproduced with permission from [82], copyright 2013 Desalination and Water Treatment.

This method, accompanied by other methods, can also be used in membrane manufacturing. In this regard, the spray-assisted LBL method is employed to produce a thin layer of polyelectrolyte/CNT [82]. A 20% solution of MWCNT/ethanol that has been ultrasonicated for 30 minutes is added to an aqueous poly(sodium 4-styrenesulfonate) (PSS) solution to produce a homogeneous PSS solution. Furthermore, poly(diallyldimethylammonium chloride) (PDDA) aqueous solution is made by adding the polymer to deionized water. Both resulting solutions are strong polyelectrolytes and can be ionized in a wide range of pH. Before producing the film, the polyestersulfone (PES) substrates are soaked in 25 °C water for 24 hours and the water is replaced every three hours. Then the spraying process is carried out with a 0.35 mm nozzle under 20 psi pressure. By repeating this process, a thin film of PSS/MWCNT-PDDA is formed on the PES substrate. In this process, the deposition of PSS/MWCNT on the PES substrate was initiated by hydrogen bonds and hydrophobic interactions and the positively charged PDDA bonds with the PSS/MWCNT layer via electrostatic and van der Waals forces. This scheme is illustrated in Figure 12.

#### Inert gas flow

**Figure 13:**
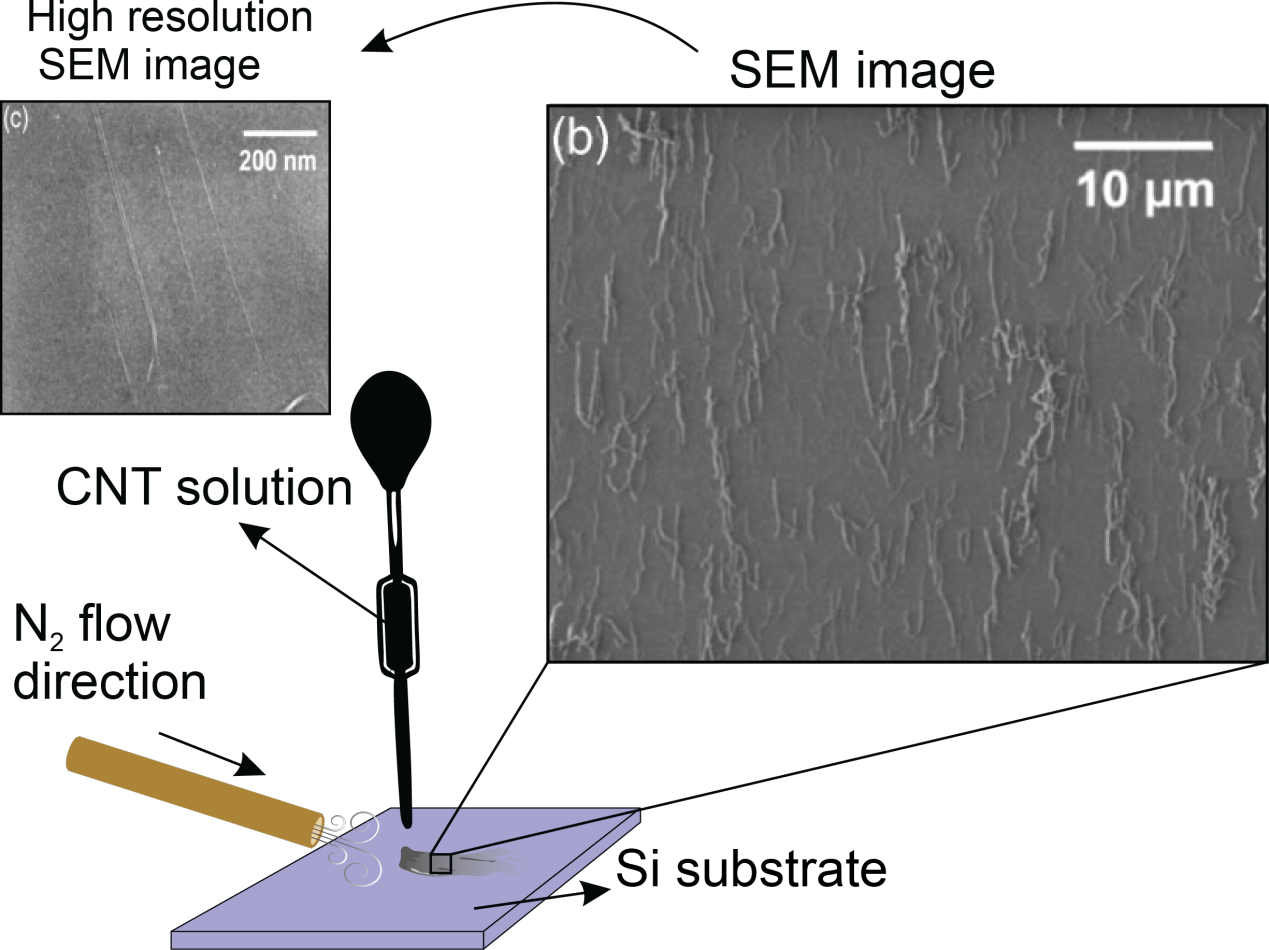
Schematic setup of the gas flow orientation system including a pipette to distribute the CNT solution and a nozzle to control the N_2_ flow direction on the Si substrate. SEM image of aligned CNTs is reproduced with permission from [95], copyright 2005 American Institute of Physics.

In this method, the CNTs are arranged by flowing a gas along the substrate. The CNT suspension is deposited drop-by-drop on a substrate and an inert gas, with a linear velocity of approximately 10 cm/s, and flows along the substrate simultaneously. The gas flow concurrently spreads the droplets and also orients the CNTs in the direction of the gas flow (Figure 13). This simple method can automatically cover a wide area of the substrate [95].

#### Liquid crystal molecules

**Figure 14:**
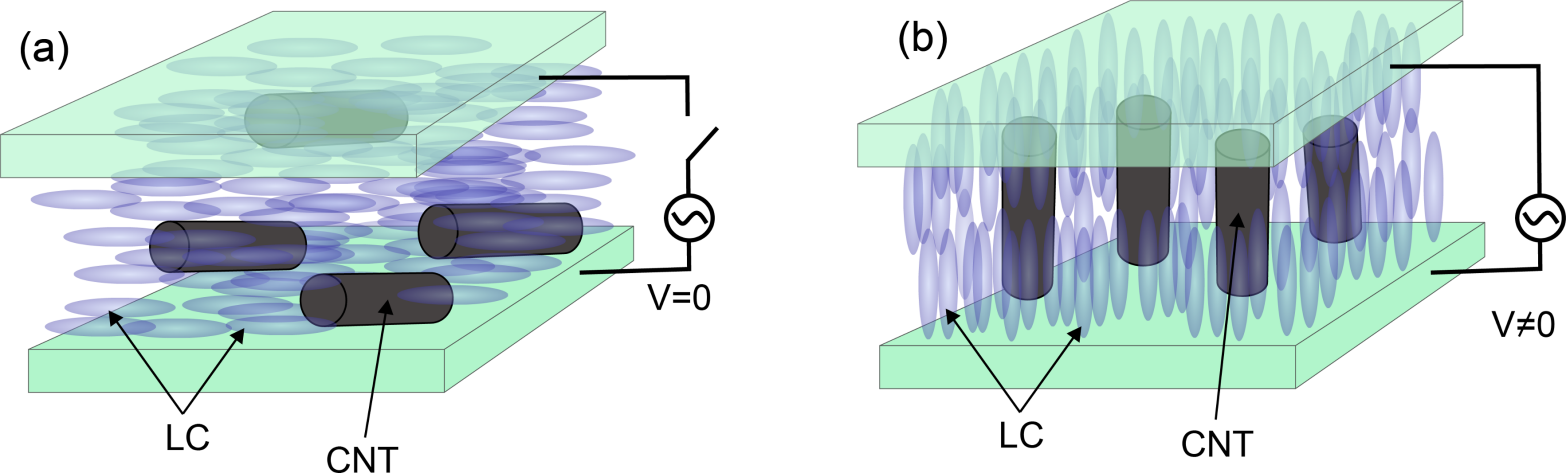
Schematic structure of a liquid crystal (blue ellipsoids) and CNT (black cylinders) rearrangement under an external electric field (a) *V* = 0 (b) *V* ≠ 0.

When a solid is heated to its melting point it loses its molecular order and is converted to a liquid with molecules in random orientation. When heated, some materials such as cholesteryl benzoate first convert to a liquid crystal (LC) phase and then convert to a liquid. Liquid crystals are made of rod-shaped molecules that are aligned parallel to each other and show different properties in different directions (Figure 14). For example, they become transparent at high frequency and opaque at low frequency.

**Figure 15:**
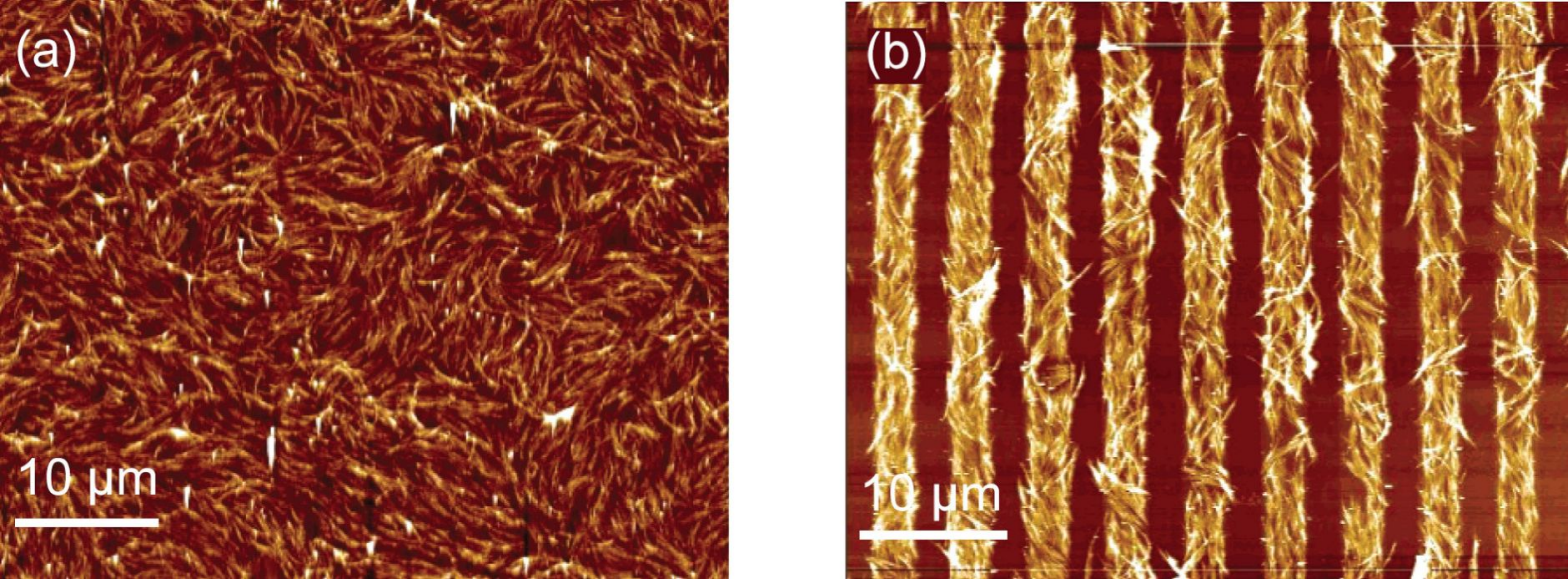
AFM images of a CNT-thin-film transistor with (a) randomly and (b) well-oriented CNT arrays, adapted with permission from [98], copyright 2006 American Chemical Society.

Because of the unique molecular structure of the liquid crystal (LC) (i.e., that it can be simply oriented in the direction of an applied electric or magnetic field) the alignment and dispersion of the CNTs in a solution of liquid crystals is also achievable (Figure 15). The biggest advantage of this method is that small fields are required to align CNTs as compared to cases where liquid crystals are not used. However, difficulties in the preparation of LC phases, especially at high concentrations, limit its applications [96–98]. On the other hand, it is known that progress in CNT alignment using this technology depends more on the quality of the CNTs than the ability to control the interactions of the CNT and LC [84].

#### Filtration method

**Figure 16:**
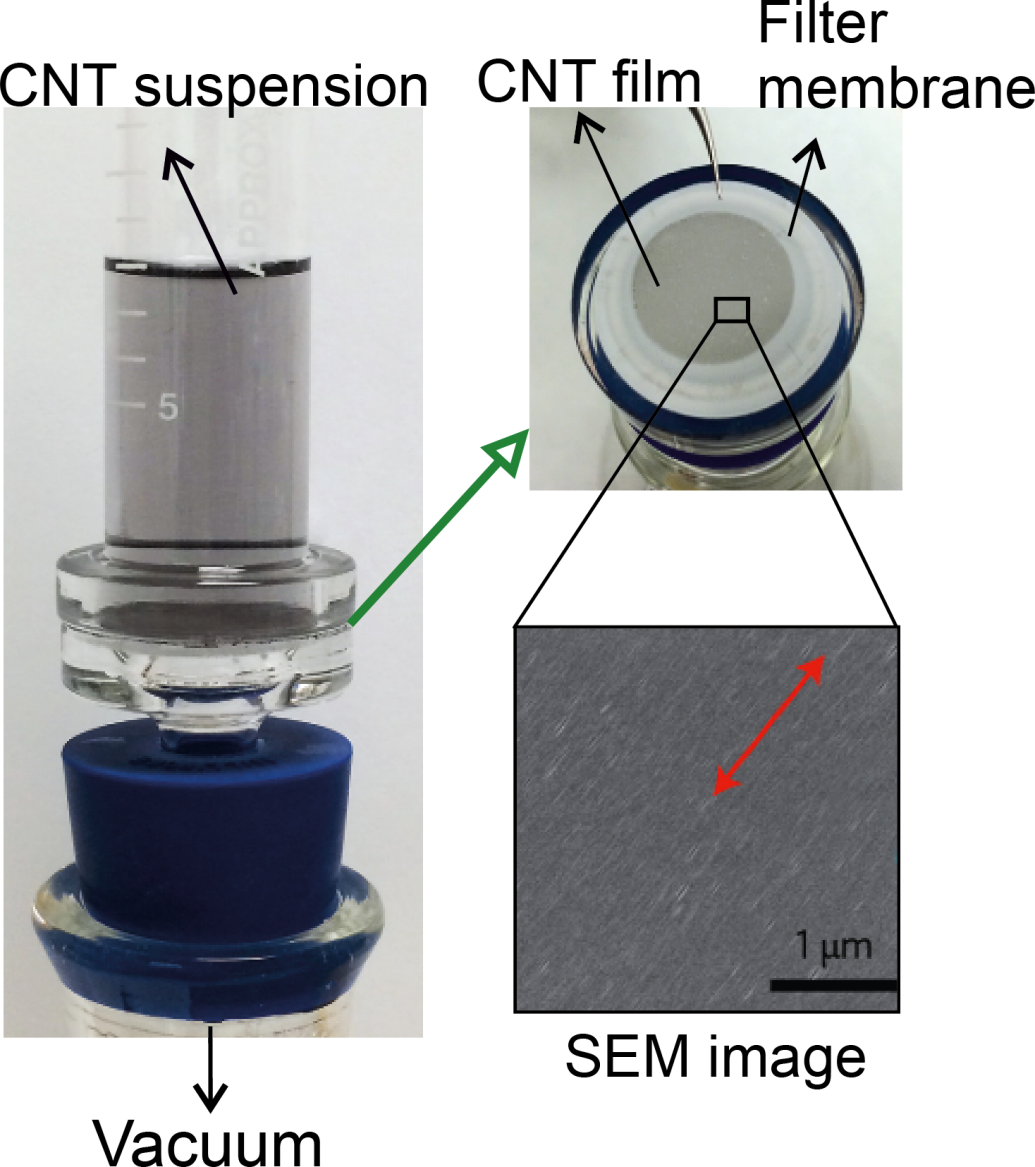
The filtration apparatus to fabricate the aligned CNT film on the membrane surface. The SEM image confirms the alignment of the CNTs, adapted with permission from [99], copyright 2016 Nature Publishing Group.

Filtering a well-dispersed and dilute solution of CNTs through a porous membrane may lead to the arrangement of the CNTs [99]. A very narrow pore size distribution (a thousand nanometers, approximately) in the membrane structure is needed to achieve a significant arrangement, which makes the process slow and inefficient. Furthermore, large masses of CNTs may quickly block the pores, causing some non-arranged CNTs to be transferred onto the substrate [100]. Nevertheless, combining the properties of LCs (as mentioned in the previous section) with the filtration method was found to effectively enhance the alignment [101]. Figure 16 shows a filtration apparatus to fabricate the aligned CNT film on the membrane surface.

#### Forming thin carbon nanotube layers by the pulling procedure

**Figure 17:**
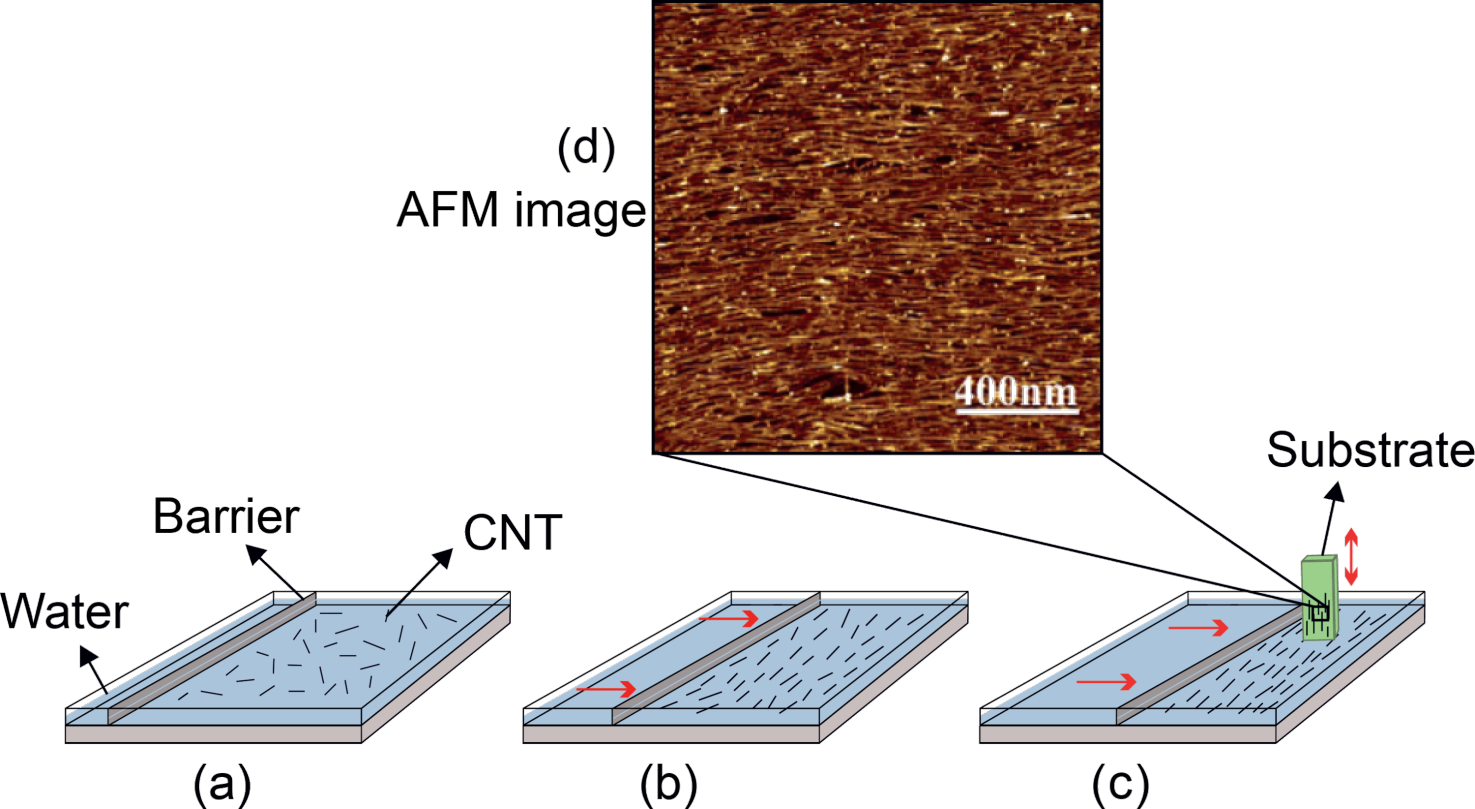
Scheme of the Langmuir–Blodgett technique: (a) the CNT suspension in the LB device, (b) the preparation of films by barrier compression, (c) substrate dipping vertically, and (d) atomic force microscopy (AFM) images of the aligned CNTs, adapted with permission from [105], copyright 2007 American Chemical Society.

The method is illustrated schematically in Figure 17. The main step in this method, known as the Langmuir–Blodgett (LB) technique, is to immerse a solid substrate into a well-dispersed CNT solution and slowly and gently pull it out (≤1 cm/min). The result is the formation of a thin homogeneous layer of CNTs oriented in the immersing direction. The three factors controlling the thickness of the layers are the CNT concentration in the solution, the number of dips and the speed by which the substrate is pulled from the solution. The alignment of the CNTs, in this method, is achieved as a result of capillary forces. Although the method is slow, because it supports a wide variety of substrates and the whole process is automatic, it is an appropriate method for industrial applications [7,102–104].

#### Acoustic waves

**Figure 18:**
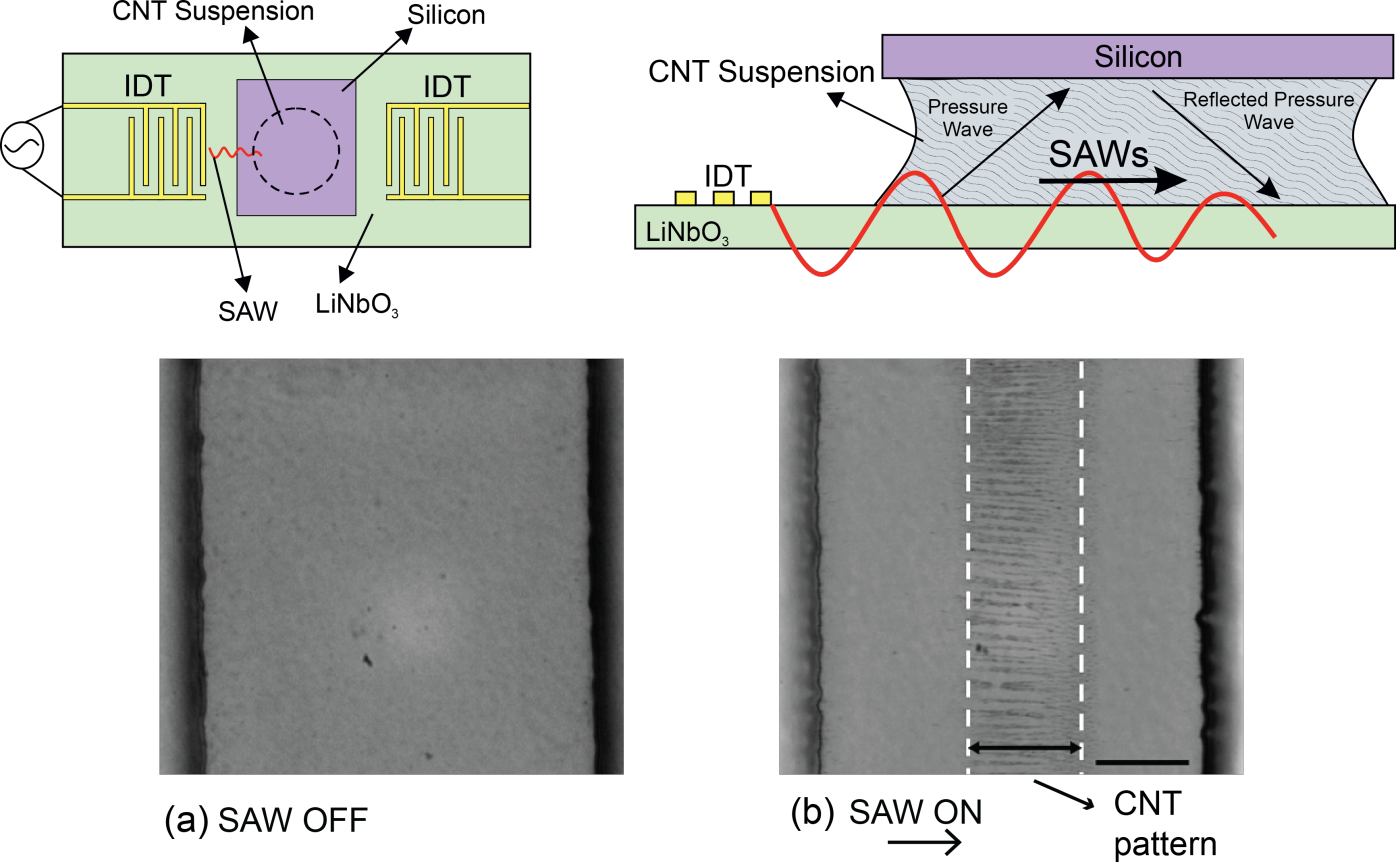
Schematic view of the experimental setup for surface acoustic wave (SAW)-based CNT arrangement. (a) Micrographs of the CNT suspension before applying the SAW field and (b) applying the SAW field and formation of the CNT patterns. Scale bar is 50 μm. Optical microscope images, reproduced with permission from [106], copyright 2013 AIP Publishing LLC.

In this method, surface acoustic waves (SAWs) are used to align and orient CNTs. SAWs are produced by applying a suitable electric field to a piezoelectric material such as LiNbO_3_. One set of metallic interdigital transducers (IDTs) intercalated on the piezoelectric surface introduces the electric field, generating a SAW displacement amplitude on the order of 10 Å. A solution of CNTs, produced by using a surfactant such as sodium dodecylbenzene sulfonate (SDBS), is dropped on a thin silicon layer that has gap-cell electrodes. An acoustic field is applied to the drops and aligns the CNTs (Figure 18). An electrical circuit is completed by creating a bridge of CNTs between the electrodes. This method is also used to produce electrical contacts with individual CNTs [28,106–107]; and recently, the ability of the technique has been investigated to purify metallic SWCNTs from the mixture species [106].

#### Magnetic field

**Figure 19:**
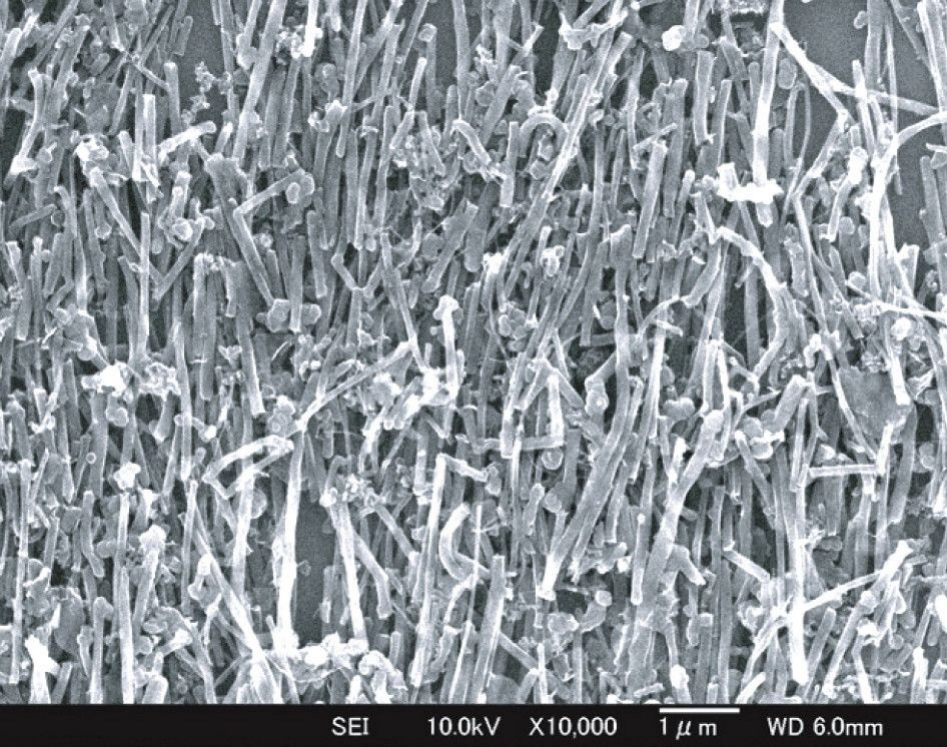
SEM image of CNTs arranged in ethanol by a magnetic field, reproduced with permission from [108], copyright 2009 Science and Technology of Advanced Materials.

The alignment of CNTs using a magnetic field is a unique technique because of its remote action. One of the most common methods to apply a magnetic field is to cast the suspension of CNTs onto a substrate that is placed in the vicinity of a magnet. While the layer on the substrate is drying, CNTs are aligned in the direction of the magnetic field. In this case, unlike an electric field which moves the CNTs, the magnetic field only reorients them. This method is not limited to CNTs and can be used to align any carbon fiber. Figure 19 indicates the SEM image of the arranged CNTs in ethanol. In this study, the magnetic field magnitude was 10 T [108].

Although magnetic field alignment seems to be ideal, due to the weak magnetic properties of CNTs, it postulates a very strong magnetic field of ≥7 T. This is why samples are usually placed inside a very narrow tube of a magnetic superconductor. Assembling CNTs with magnetic nanoparticles (MNPs) or packing them with LC molecules may reduce the required magnetic field [108–110]. Aleman et al. have recently reported the ferromagnetism of residual catalysts in CVD growth of CNTs. This work shows that adjustment of the size and shape of the catalyst nanoparticle can control the CNT ferromagnetism behavior. This phenomenon can be effective in developing the method [111].

The magnetic field strength and sample size are the limiting parameters of this method. Moreover, as previously mentioned, the CNTs are aligned in the direction of the axis of electrospun nanofiber polymers. In new research, well-aligned electrospun nanofibers containing MWCNTs were successfully fabricated by a magnetic field [110].

#### Electric field

The alignment and orientation of CNTs by an electric field is applied in two ways: as electrophoresis (EP) and dielectrophoresis (DEP). EP is the transport of charged particles through a medium enforced by a uniform electric field. This method has some limitations as the particles must be charged. DEP is also a phenomenon related to electrophoresis but with some important differences. It uses a nonuniform electric field to enforce uncharged particles to move. In this method, small droplets of a CNT suspension are placed on a substrate that has some interdigitated electrodes. After applying an AC electric field, the CNTs are aligned between the electrodes. This product is widely used in electrical CNT equipment. A DC electric field is not suitable because it causes the accumulation of CNTs near one of the electrodes. Another advantage of this method is the possibility of separating metallic CNTs (m-CNTs) and semiconducting CNTs (s-CNTs). Because of different the responses to an electric field, m-CNTs are attracted to the electrodes while s-CNTs are eliminated from the substrate by the flow of fluid.

**Figure 20:**
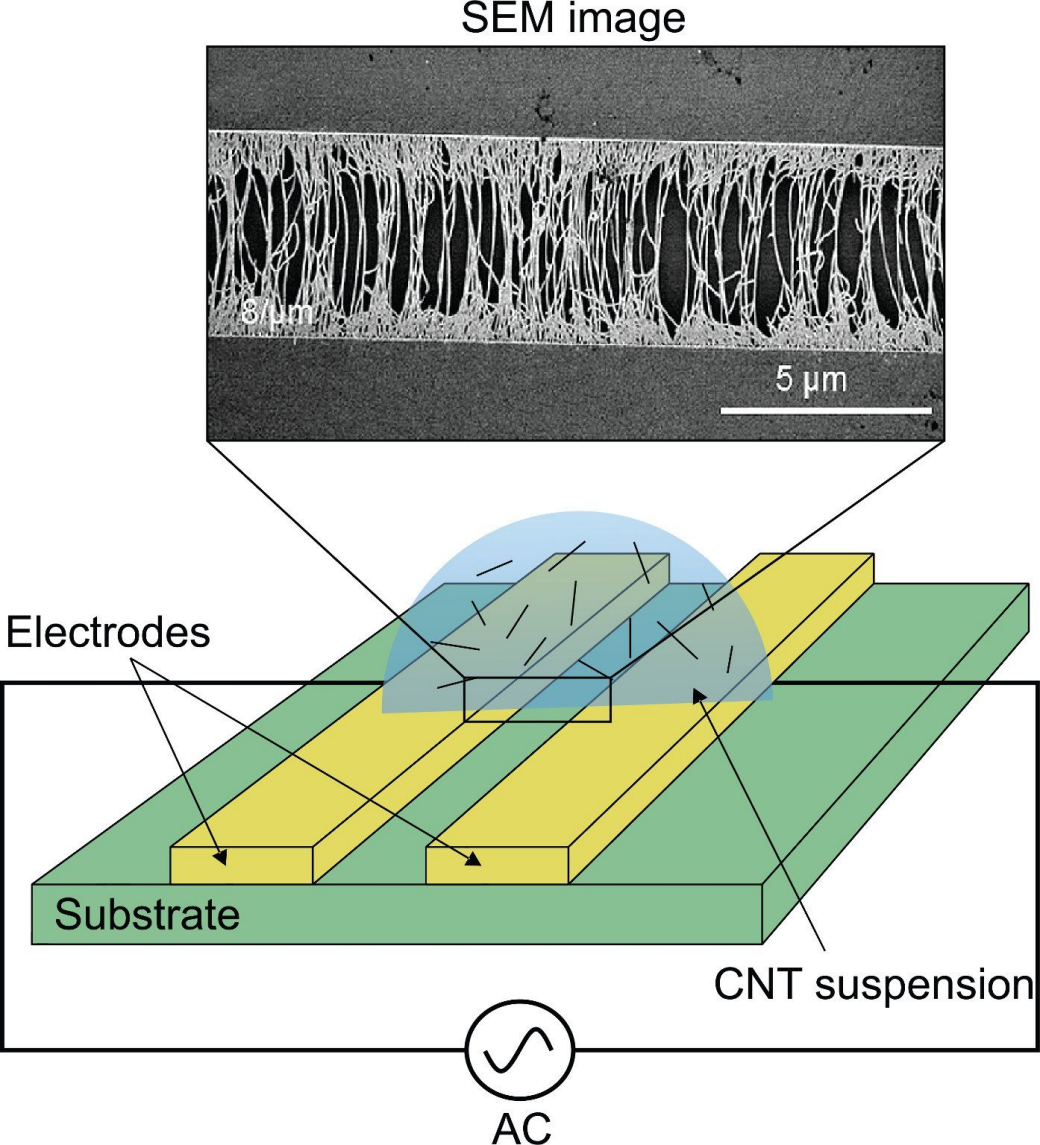
Schematic of a general dielectrophoresis (DEP) system to fabricate the oriented CNT patterns by an AC electric field. The SEM image of the aligned CNTs adapted with permission from [115], copyright 2011 American Chemical Society.

Although this method is simple, its success depends on many factors including the CNT concentration in the solution, the electric field strength (103 V/cm) and its frequency (kHz–MHz) [112–115]. A system of aligning SWCNTs using an AC electric field is shown in Figure 20.

#### Other techniques

Besides the above techniques summarized in Table 1, there are other less widespread methods such as the dip-pen nanolithography technique [116], uniaxial pressure technique [117–118] and the use of femtosecond laser pulses [119], which are not presented here.

**Table 1:** A summary of the discussed techniques for the orientation and arrangement of carbon nanotubes.

Technique	Effective force^a^	Phase application^b^	Advantage	Restriction	Ref.

stretching	M	S	high degree of alignment and high production rate	scale-up	[33,38]
fracture	M	S	simple setup	not easy to control	[6,27–29,31–34,38–39]
doctor blade (DB)	M	S	simple setup, ability to use various substrates	destructive	[41–42]
filtration	M	L	simple setup	slow, inefficient	[100]
electrospinning (ES)	E	S	simple setup, high degree of alignment, fast	unusable for all polymers, effectiveness depends on quality of CNT dispersion	[45–47]
layer-by-layer (LBL)	M/Ch	L/S	flexibility combined with other methods, widespread usage of materials in various shapes	compatibility of materials used as varied layers	[40,78–83,93–94,103]
gas flow	M	L	simple setup	scale-up	[95]
Langmuir–Blodgett (LB)	M/Ch	L	flexibility combined with other methods, useful for fabrication of electromagnetic devices on a large scale	effectiveness depends on CNT suspension and minimization of intermolecular interactions	[7,102–104]
acoustic waves	M	S	ability to scale up for fabrication of large-area planar structures and composite material, fast	limitations on the type of materials (low viscosity thermosets)	[28,106–107]
magnetic field	MG	L/S	real-time manipulation and engineered patterning of CNTs, high degrees of orientation, use in electronic devices	small sample, requires high magnetic field, hindrance in industrial mass production	[108–110]
electrophoresis (EP)/dielectrophoresis (DEP)	E	L/S	real-time manipulation and engineered patterning of CNTs, high degrees of orientation, use in electronic devices	small sample requires high voltage	[112–115]
liquid crystal (LC)	M/Ch/E/MG	L	requires small fields for orienting CNTs, use in electronic devices	unusable for various materials, depends on CNT quality	[96–97,120]

^a^M = Mechanical, Ch = Chemical, E = Electrical, MG = Magnetic; ^b^L = Liquid, S = Solid.

### Evaluating the arrangement and alignment of CNTs

After describing the techniques that can be implemented to align CNTs, understanding the methods for evaluating and characterizing them is necessary. These methods are, based on the nature of identification, classified into three main groups: microscopic analysis methods, phase analysis methods and surface analysis methods.

#### Microscopic analysis methods

The aim of these methods is to create magnified images of the material. The resolution is determined according to the lowest achievable concentration of rays. For example, a resolution of about 1 μm and of about 1 Å is achievable by using optical and ion microscopes, respectively. In analyzing the alignment of CNTs, the most common microscopic methods are atomic force microscopy (AFM), transmission electron microscopy (TEM), scanning electron microscopy (SEM) and scanning transmission microscopy (STM).

**Electron microscope:** By changing the curvature and number of lenses (concave or convex) in the optical microscope we can enlarge the images; but images at a magnification of higher than 2000 lose resolution due to the long wavelength of light. The resolution is the shortest distance between two points that can be distinguished as separate points.

An electron microscope uses a beam of electrons instead of light. Because the electron wavelength is very short, images can be magnified up to a million times or more in some electron microscopes. However, using electron beams creates certain constraints. The first limitation is that images are in black and white because, unlike light, the electron beam does not carry color information. However, in modern in systems with image analysis software, a pseudo-color image can be obtained by adding artificial colors to the grayscale image. The second limitation is that, unlike light, electrons cannot easily move in the air; therefore, a very strong vacuum is needed along the path of the electrons and also in the sample chamber. Vacuum is usually created by using a rotary pump and a diffusion pump. The complementary information is available in detail in reference books [121–124].

Three types of electron microscopes are used in analyzing the alignment and arrangement of CNTs. The first type is a scanning electron microscope (SEM), in which electrons are emitted and reflected from the surface of the sample, then they are collected by the detectors and converted to photons of visible light to create a visible image. These images offer useful qualitative information about the placement and alignment of CNTs, the diameter distribution and their relative purity [125].

The second type, TEM, is one of the most useful and important techniques used in research on CNTs. In this method, the size and shape of particles are determined by a resolution of a few tenths of a nanometer, which depends on the type of material and equipment that is used [124]. Nowadays, high-resolution TEM (HR-TEM) is used in analyzing properties of nanostructured materials.

The third type, AFM, is a technique that is used to study the structure and properties of materials at the nanometer scale. Flexibility, having multiple potential signals, and the capability of operating under various conditions have enabled researchers to examine a wide variety of surfaces under different environmental conditions. Furthermore, this technique can work in a vacuum, air, and liquid environments. Unlike other methods of analyzing surface properties, most of the time there is no fundamental limit to the type of surface and environment in this method. With this device, it is possible to analyze conductive or insulating, soft or hard, solid or powder, biological, and organic or inorganic surfaces. This device can measure geometric morphology, adhesion distribution, friction, surface impurities, texture, elasticity, magnetism, chemical bonding forces, distribution of electric charges and electric polarization in different parts of the surface. In practice, this feature is used to study corrosion, cleanness, uniformity, roughness, adhesion, friction, size, etc. AFM, like SEM and TEM, is a suitable technique to characterize the alignment of CNTs, especially horizontal alignment; for instance, when the CNTs grow horizontally on a substrate made of quartz [126].

#### Phase analysis methods

In these methods, the crystal structure or the minerals in the material are identified. For example, the type and percentage of oxides contained in a sample can be identified and measured. The most famous phase analysis method is X-ray diffraction (XRD) spectroscopy. XRD is used to determine most of the properties of a crystal structure such as lattice constants, lattice geometry, recognition of unknown materials, crystalline phases, size of the crystal, single crystal orientation, stress and lattice defects [127–129]. This method is based on the fact that X-rays are electromagnetic waves with a wavelength on the order of 5.0 to 2.5 Å.

**Figure 21:**
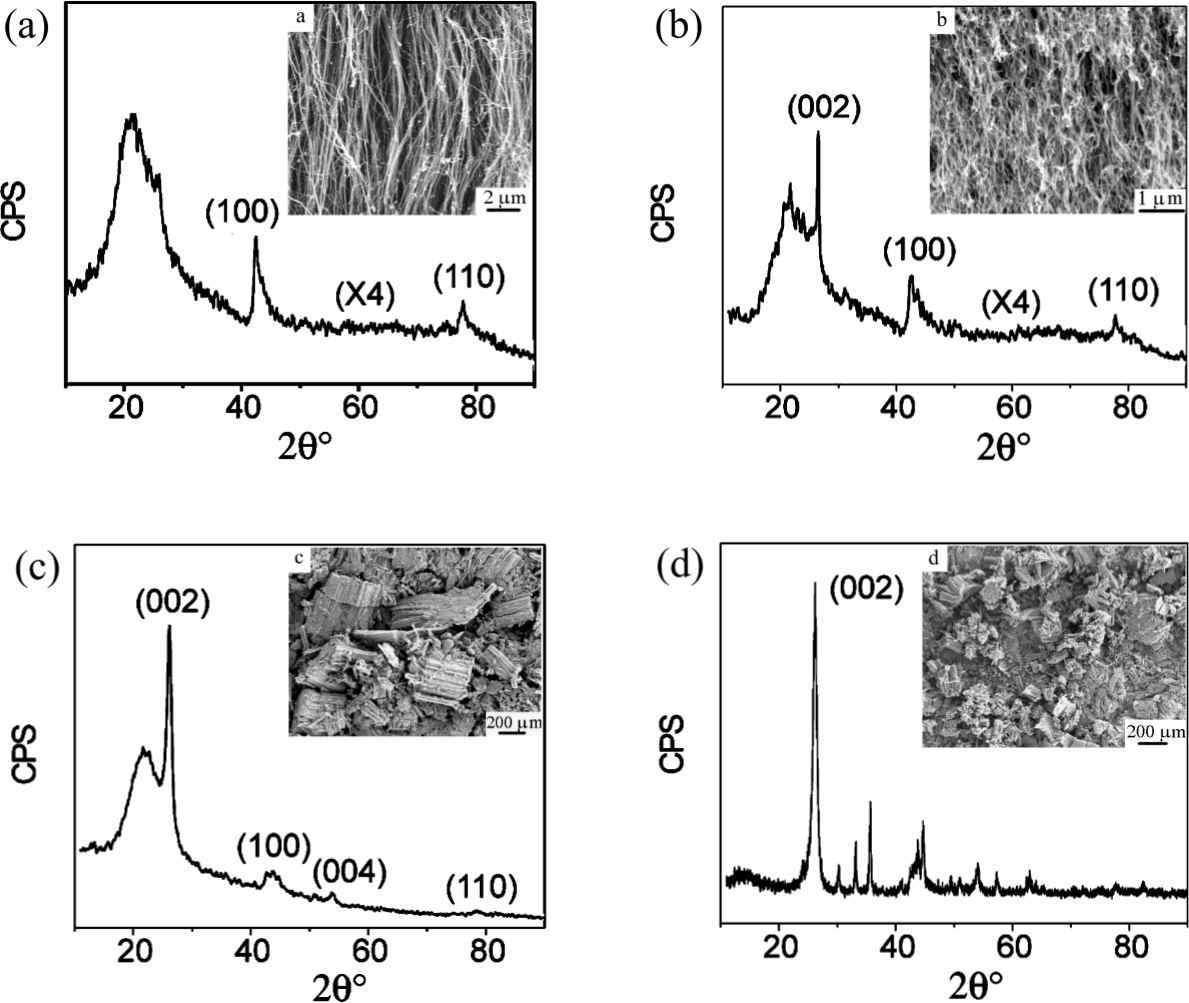
XRD results and SEM images of CNTs with various degrees of alignment: (a) as-grown straight CNT arrays with good alignment, (b) as-grown curled CNTs with less alignment, (c) disordered CNT arrays, (d) disordered CNT arrays. The substrate causes the broader peak at 20° and the intensities in (a) and (b) to be increased by times. Reproduced with permission from [130], copyright 2001 Elsevier.

2D detectors are very helpful to record XRD patterns. Cao et al. have shown that the degree of CNT alignment can be determined by the peak intensities in XRD patterns. Figure 21 shows four samples with different alignments and their XRD results. It indicates the intensity of the (002) peak is enhanced if the CNTs in the sample are less aligned [130].

Small-angle X-ray scattering (SAXS) is a method for evaluating the particle size distribution or nanohole size distribution in the sample. This sample can be amorphous, crystalline or a macromolecule (e.g., polymer molecule). SAXS provides information about electron density fluctuations that occur in the sample that is calculated by analyzing the intensity of scattered X-rays with a scattering angle of 0.1–5°. The local inhomogeneity in amorphous materials, colloidal particles, and agglomerated particles can be identified by this method.

Also, long-range order and the distance between the particles in a collection of polymer molecules can be determined by using SAXS and structural models. This method is non-destructive and can be used for analyzing samples that are not transparent to visible light.

SAXS can identify particles or holes between 1 to 100 nm. Accurate determination occurs for a particle size of 1 to 10 nm size; however, it can also be used for particles with sizes outside of this range. The average size of the particles or holes can be obtained by the shape. By using SAXS, the surface-to-volume ratio can also be determined. In the case of CNTs, SAXS provide both quality and quantity information on the nanoscale, such as average diameter and orientation, respectively. This is due to the mesoscopic size of the X-rays, which is typically on the order of a few hundred micrometers. This dimension is small relative to the typical millimeter scale of the whole aligned CNT sample and large in relation to individual CNTs on the nanoscale. Thus, a small region of the CNTs can be probed by SAXS beams to get some information about millions of CNTs. The mesoscopic size scale of scattering techniques has been used to reveal information about the alignment of CNTs in CNT fibers [131], the average diameter and orientation of CNTs grown on substrates [132–133], dispersion of CNTs in liquids [134], and the sorting of CNTs in CNT bundles [135].

#### Surface analysis methods

The surface of a solid does not present the same chemical condition as compared to the bulk because of its connection with the surroundings. The surface plays an important role in many applications and processes, especially in alignment and arrangement of CNTs. Also, the chemical composition of the surface is different in the bulk due to the placement of functional groups on the CNT surface. Thus, generalizing the chemical analysis results of a bulk sample from the surface analysis would not be accurate. On the other hand, bonding at the CNT/polymer interface (covalent, van der Waals) also plays a decisive role to enhance the uniformity of the CNT dispersion into the polymer matrix [136]. Therefore, to obtain the desired properties of CNT/polymer composites, such as the thermal, mechanical and electrical properties [8,10,137], the characterization of surface properties including the CNT surface orientation and surface defects are also important [138–140]. The defects can have both positive (increasing the bonding at the interface and between carbon structures [25,137,139,141]) and negative (reduction of the quality of physical properties [10,141]) effects on the application and quality of composites.

For this aim, many techniques have been developed such as Raman spectroscopy, Fourier-transforms infrared spectroscopy (FTIR) and X-ray photoelectron spectroscopy (XPS).

**Figure 22:**
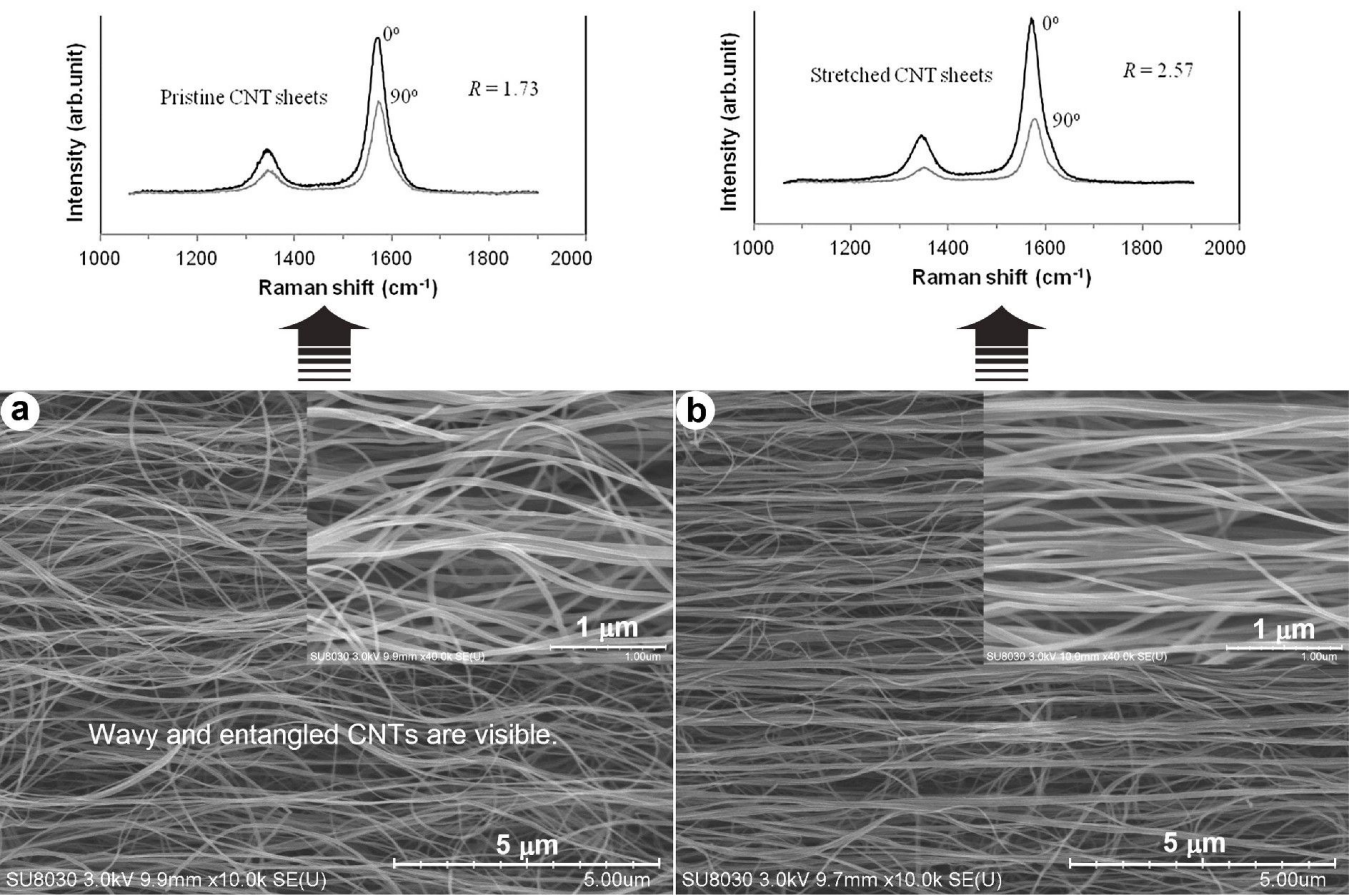
FE-SEM micrographs presenting the microstructural morphology of (a) random and (b) aligned CNT sheets with polarized Raman spectra at 0° and 90°. Reproduced with permission from [142], copyright 2016 Elsevier.

**Raman spectroscopy:** Raman spectroscopy is a powerful tool to identify and quantify samples. This method gives significant information about molecular vibrations. The technique involves the excitation of a sample with a monochromatic light source (i.e., laser) and collecting the scattered light. The Raman spectra of CNTs can be identified with the radial breathing mode (RBM), tangential mode (G-band), disorder-induced mode (D-band), and other Raman features, determining the physical properties of the material. The G-band intensity as a function of the angle of polarization for individual CNTs can be related to their arrangement so that the G-band intensity of well-aligned CNT bulk materials should be higher than that of less-aligned CNTs. Figure 22 shows field emission (FE)-SEM micrographs of two CNT samples with random and aligned orientation and their polarized Raman spectra at 0° and 90° (0° related to the polarization direction of the laser light where it is parallel to the CNT alignment direction, and 90° related to the laser light polarization direction where it is perpendicular to the CNT alignment direction). As indicated in the graph, the ratio of the G-mode to the D-mode (*R*) has increased [142].

The composites analyzed by Raman spectroscopy typically have very strong CNT signals that mask the signals of other organic components. In such cases, tunable UV Raman spectroscopy may be used, although this technique is rarely used.

**Fourier-transform infrared spectroscopy:** FTIR is often used to characterize molecular bonding on the CNT surface and to determine the modification of the CNT structure by adding compounds. It is very difficult to get a good FTIR spectrum of CNTs, thus attenuated total reflection (ATR) FTIR can be used (except with carbon crystal, when germanium is more suitable). The investigation of the intermolecular interaction between the polymer chain and CNT by FTIR can confirm the results of observations [137,143–145]. For example, it has been reported that adhesion of the polymer to the CNT leads to constrained motion of the polymer chains and simplifies the charge transfer process, which consequently improves transport properties [146]. Also, many researchers indicate that the macroscopic optical properties of CNTs depend on the tube orientation with respect to the direction of beam propagation. Thus, FTIR results can be related to the morphology of the CNT mixture such as bundling, length and straightness [125].

**X-ray photoelectron spectroscopy:** The XPS technique does not explore the whole sample volume but rather provides information about the chemical composition of the CNT surface [144–145]. XPS suffers from surface charge artifacts, so correct sample preparation is crucial to achieve a flat and uncontaminated surface. Despite the mentioned limitations of this method and its ineffectiveness in assessing CNT orientation, invaluable surface information can be obtained about superficial functional groups, gases adsorption, structure modification process and defects on the CNT walls. This information is very critical to judge the final composite behavior [139,145,147].

## Conclusion

The arrangement and sorting of CNTs is an important factor in engineering and design of nanocomposite materials with desired structures. This article reviews the latest methods that have been proposed to arrange and align CNTs after growth in the structure of the composite materials, emphasizing the type of process. In all methods, CNTs are initially added to the composite and are then subjected to sorting processes using special techniques. The effectiveness of each technique on each of the properties is different, for example, the sorting method may improve the mechanical properties of the composite while its electrical and chemical properties change negligibly. Therefore, selecting an appropriate CNT orientation method in order to modify the material structure depends on the final field of composite application. In addition to the mentioned limitations for each of these methods, one of the big challenges is the need of pretreatment to homogenize the CNTs in terms of their physical characteristics such as diameter, length, number of walls and metallic or semiconducting properties. In fact, the selected pretreatment method can affect all of the CNTs for maximum controllability. In this regard, it seems that focusing on the principles and basic concepts of the CNT orientation mechanism in the composites is essential as well as CNT separation based on geometrical factors.

The effect of parameters such as optimal time to end the process, concentration, interactions of CNTs with polymeric and metallic materials, impact of internal and external CNT walls and the effect of physical properties, such as chirality, length and diameter on alignment needs to be understood. Furthermore, to achieve uniform composite structures, the separation of metallic and semiconducting CNTs is essential before alignment of CNTs. Considering the latest research carried out in this field, it has been indicated that using an electric field combined with mechanical methods would improve the separation of metallic and semiconducting CNTs. However, we are still a long way from achieving an easy, inexpensive, fast and highly applicable process for comprehensive use of CNTs in new composite industries.
